# Interfacial charge transfer processes in 2D and 3D semiconducting hybrid perovskites: azobenzene as photoswitchable ligand

**DOI:** 10.3762/bjnano.11.38

**Published:** 2020-03-17

**Authors:** Nicole Fillafer, Tobias Seewald, Lukas Schmidt-Mende, Sebastian Polarz

**Affiliations:** 1 University of Konstanz, Universitätsstrasse 10, 78467 Konstanz, Germany; 2 Leibniz-University of Hannover, Institute of Inorganic Chemistry, Callinstrasse 9, 30167 Hannover, Germany

**Keywords:** interface design, molecular switches, organic–inorganic hybrid materials, particle synthesis, semiconductors, transport across interfaces

## Abstract

In the vast majority of studies on semiconductor particles ligands or capping agents are used that bind to the surface of the particles covering them with an electrically insulating shell. Since the transport of charge carriers and/or energy across interfaces is desirable for a variety of applications, the use of π-conjugated ligands becomes increasingly interesting. Among them are compounds that react to external stimuli. Molecular switches in particular are fascinating because the properties of the interfaces can be potentially adjusted as required. However, there is debate about how the properties of such special ligands are influenced by the presence of a semiconductor and vice versa. Here ammonium-modified azobenzene compounds were selected as prototypes for molecular switches and organic–inorganic hybrid perovskites as semiconductor materials. The class of ammonium–lead–halide phases as prototypes is peculiar because, in addition to the surface functionalization of 3D crystals, organic compounds can actually be incorporated into the crystal as 2D phases. Thus, for example, layered Ruddlesden–Popper phases are obtained. We present photoswitchable azobenzene ligands with different head-group lengths for the synthesis of 2D and 3D hybrid perovskite phases. The energy transfer mechanisms are influenced by the length of the molecular spacer moiety, which determines the distance between the π system and the semiconductor surfaces. We find huge differences in the photoswitching behaviour between the free, surface-coordinated and integrated ligands between the perovskite layers. Photoswitching of azobenzene ligands incorporated in 2D phases is nearly quenched, while the same mechanism for surface-coordinating ligands is greatly improved, compared to the free ligands. The improvement originates from an energy transfer from perovskite to azobenzene, which is strongly distance-dependent. This study provides evidence for the photoswitching of azobenzenes as ligands of hybrid perovskites, which depends on the spacing between the chromophore and the perovskite phase.

## Introduction

Recently the class of hybrid perovskites attracted great attention in materials chemistry and physics [1–3]. In addition to an outstanding performance in photovoltaics, a peculiar feature is that not only compounds with a three-dimensional (3D) expansion of the crystal lattice exist. Also, a distinctive structural feature enabling the formation of low-dimensional phases provides a versatile way for the development of novel functional materials [4–6]. Higher stabilities compared to the 3D phases make these 2D phases a promising material for optoelectronic applications [7–11]. Larger organic cations “cleave” the crystal structure of the perovskite and are integrated into the material, yielding a high degree of chemical diversity to this class of organic–inorganic solids. Dissection of the perovskite structure along certain crystal facets ((100), (011), (111)) leads to low-dimensional (2D) and quasi-2D phases, which are known as Ruddlesden–Popper phases [4,12]. From a chemist’s view this opens up the possibility to add novel functional cations not only to the surface, but also to the inner structure of the materials [13]. Of course, the molecule must not be too sterically demanding, otherwise it could not be incorporated [14]. Taking these conditions into account, a number of organic cations have so far been incorporated into the perovskite structure [15].

For the present work the combination of a lead-containing hybrid perovskite with π-conjugated organic molecules is of importance. Functionalizing hybrid perovskites with conjugated π systems improves not only the optoelectronical properties and stability but as well the conductivity [16–20]. In 1997, Era et al. first presented a chromophore-containing organic–inorganic perovskite [21]. They observed an enhanced phosphorescence of the included naphthalene [22], which was explained by an efficient energy transfer from Wannier excitons from the semiconducting perovskite layer to the triplet states of naphthalene [23]. Regarding the electronic alignment of molecular orbitals, valence and conduction bands, the chromophore may serve as a quantum well [24]. The energy transfer between the semiconducting perovskite layer and the chromophore can easily be influenced by the chemical structure and design of the organic cations [25–26].

A number of molecular π systems contain a stimuli-responsive moiety. These compounds have been widely discussed in literature as molecular switches [27–29]. Among these, azobenzene derivatives are very prominent and have been studied extensively [30–32]. The extended aromatic structure, comprising two phenyl rings connected through an azo group, shows characteristic and unique photoswitching properties. Absorption of a photon in the UV region causes an isomerisation of the nitrogen–nitrogen double bond. The conformational change is accompanied not only by a length variation but also by a significant change of the dipole moment [33–34]. Azobenzenes were used in various materials namely as liquid crystals, optical switches or for data storage [35–36]. Because of the chemical and physical changes associated with the switching process, the combination of azobenzene molecules with conducting or semiconducting materials is of high interest. In connection with semiconductors the occurrence of interesting phenomena has been proposed. The symmetry-allowed π→π* transition and the subsequent structural relaxation can be affected by the electronic system of a semiconductor. Torres et al. reported calculations of a charge-transfer complex of para-methyl red/TiO_2_ in the gas phase where the isomerisation is fully quenched. The photoexcited state is oxidized immediately through a charge transfer from the azobenzene to TiO_2_ and no conformational change can be observed [37]. Other findings show, that the direct linkage to a semiconductor may even improve the photoswitching reaction through electron injection from the semiconductor to the azobenzene [38].

Based on the above arguments, it seems to be very tempting to combine π-conjugated photoswitchable compounds of the azobenzene type with hybrid perovskites and incorporate them as a conductive phase. There are a few publications on 2D layered hybrid perovskites (LHPs) containing azobenzene [21,39–40]. Here, the cation used serves only as structuring chromophore but not as an electronic part of the materials. Sasai et al. were the first to observe a photoswitching reaction between the perovskite sheets using a combination of UV–vis irradiation and photoluminescence [41]. However, in particular the aspect of photoswitching of azobenzene–perovskite hybrids is still very unclear. For this reason, we present here an extended series of experiments that deal with this issue. First, we present the synthesis and characterization of a collection of azobenzene molecules that differ systematically in the length of a linker between the π-conjugated photoswitchable part and the ammonium group, which is responsible for interaction with the perovskite phase. Next, we describe the use of these compounds during perovskite synthesis, aiming to obtain either an incorporation into the lattice (2D phases) or only a surface stabilization (3D phases). Finally, we investigate the optoelectronic properties in detail focussing in particular on photoswitching and charge-carrier transport.

## Results and Discussion

### Preparation and characterization of photoswitchable ligands

**Figure 1:**
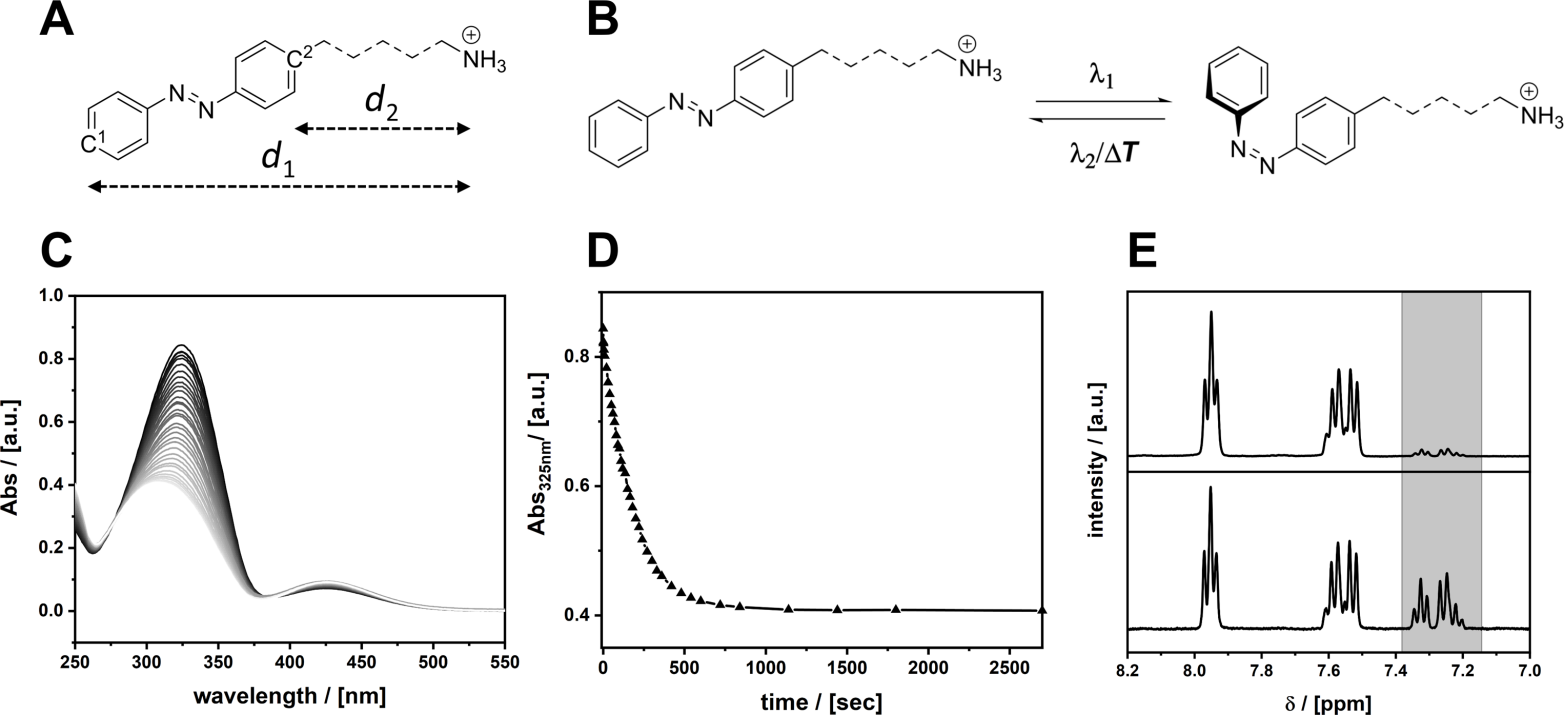
(A) Geometrical description of the azobenzene derivatives used in the current study. (B) Scheme of the photoswitching reaction of the azobenzene ligands under irradiation with UV light (λ_1_); a conformational change from *trans*- to *cis*-conformation occurs. Blue light (λ_2_) or heat (Δ*T*) cause the reverse reaction. (C) UV–vis kinetic measurement of AzoC_2_ in purified H_2_O. The solution was irradiated at 313 nm. The black line marks the beginning of the reaction, the light grey lines mark the ending of the reaction. (D) Absorption at 325 nm measured over a time period of 2600 s. (E) ^1^H NMR of AzoC_2_ in MeOD from 8.0 to 7.2 ppm, before (top) and after irradiation (bottom) at 313 nm for 4 h. Highlighted in grey is the signal of the *cis*-isomer. Measurements of the other ligands can be found in Supporting Information File 1, Figures S10–S12.

As a proof of concept four suitable azobenzene ligands were synthesized. The preparation of the compounds using standard organic chemistry reactions is described in detail in the Experimental section. All ligands provide an ammonium headgroup, which is able to coordinate to a lead halogenide-based perovskite surface [4]. The distance between headgroup and azobenzene moiety has been varied through the introduction of different spacers (see Figure 1A). A methylene (AzoC_1_) and an ethylene group (AzoC_2_) provide a short distance and connect the headgroup directly to the photoswitchable group. A butoxy (AzoOC_4_) and a dodecyloxy group (AzoOC_12_) connect the headgroup through an ether bonding and provide a longer distance. All ligands were characterized with NMR (^1^H, ^13^C) and electro-spray ionisation mass spectroscopy (ESIMS) the results of which can be found in Supporting Information File 1 (Figures S1–S4).

Several methods were used to investigate the properties of the ligands in the absence of the perovskite as reference. DFT calculations were performed to obtain information about the geometry of the molecules (Supporting Information File 1, Figure S5) the results of which are summarized in Table 1. According to these calculations, the distance between the π-conjugated part and the ammonium group ranges from 2.5 up to 17.6 Å. The position of the relative energy levels is a decisive factor for a possible energy transfer between the azobenzene compound and the perovskite matrix. Relative energies of the electronic levels of the ligands were evaluated using a combination of methods. Photon electron spectroscopy (on air) (PESA) reveal the relative energy of the highest occupied molecular orbital (HOMO) of the molecule. In combination with UV–vis measurements the higher unoccupied MOs (S_1_ and S_2_) of the molecule can be determined. The data are summarized in Table 2.

**Table 1:** Determination of the length of the azobenzene ligands (*d*_1_) and the length of the spacer between the azobenzene moiety and the ammonium headgroup (*d*_2_).^a^

	AzoC_1_	AzoC_2_	AzoOC_4_	AzoOC_12_

*d*_1_ (C^1^–NH_3_^+^)	11.1 Å	12.8 Å	16.4 Å	26.6 Å
*d*_2_ (C^2^–NH_3_^+^)	2.5 Å	3.9 Å	7.4 Å	17.6 Å

^a^The molecules were simulated using DFT calculations and the distances were evaluated with the software Jmol (see Supporting Information File 1, Figure S5).

**Table 2:** Molecular orbitals of the azobenzene ligands determined and calculated using PESA and UV–vis measurements. All spectra can be seen in Supporting Information File 1, Figures S6–S9.

	AzoC_1_	AzoC_2_	AzoOC_4_	AzoOC_12_

S_0_ (HOMO)	−5.37 eV	−5.46 eV	−5.40 eV	−5.36 eV
S_1_ (LUMO)	−2.46 eV	−2.55 eV	−2.48 eV	−2.08 eV
S_2_	−1.50 eV	−1.65 eV	−1.78 eV	−1.51 eV

It is well known, that the S_0_→S_2_ transition (a symmetry-allowed π→π* transition) is responsible for the photoswitching from *trans*- to *cis*-conformation [42]. In this region the *trans*-azobenzene shows a much stronger absorption than the *cis*-azobenzene. The S_0_→S_1_ transition (HOMO–LUMO) is a symmetry-forbidden n→π* transition and therefore shows a much weaker absorption than S_0_→S_2_. The conformational change and the associated symmetry breaking makes the transition S_0_→S_1_ more likely for the *cis*-isomer. By irradiating a solution of the ligands in an appropriate solvent at 313 nm (3.96 eV) the photoswitching can be observed via UV–vis absorption spectroscopy (see Figure 1). In the following, we only discuss the results for AzoC_2_, all other ligands behave very similarly and the data can be found in Supporting Information File 1, Figures S10–S12. A solution of AzoC_2_ in purified H_2_O (*c* = 10^−5^ M) was irradiated at 313 nm and the absorbance was measured at certain time intervals. The absorption maximum at 325 nm is assigned to the S_0_→S_2_ transition and loses intensity over the time of irradiation (Figure 1C). The signal of the S_0_→S_1_ transition at 425 nm shows much weaker absorption in the beginning, which increases over the time of irradiation. Using ^1^H NMR (Figure 1E) the degree of isomerisation (DOI) can be determined. Multiplets from 8.0 to 7.9 ppm and from 7.6 to 7.5 ppm are significant for the molecule in *trans*-conformation, whereas a multiplet from 7.4 to 7.2 ppm (highlighted in grey) belongs to the *cis*-isomer. Integration of the signals after irradiation gives a DOI of 39.4% of AzoC_2_ in solution.

### 2D LHPs with incorporated azobenzene ligands

**Figure 2:**
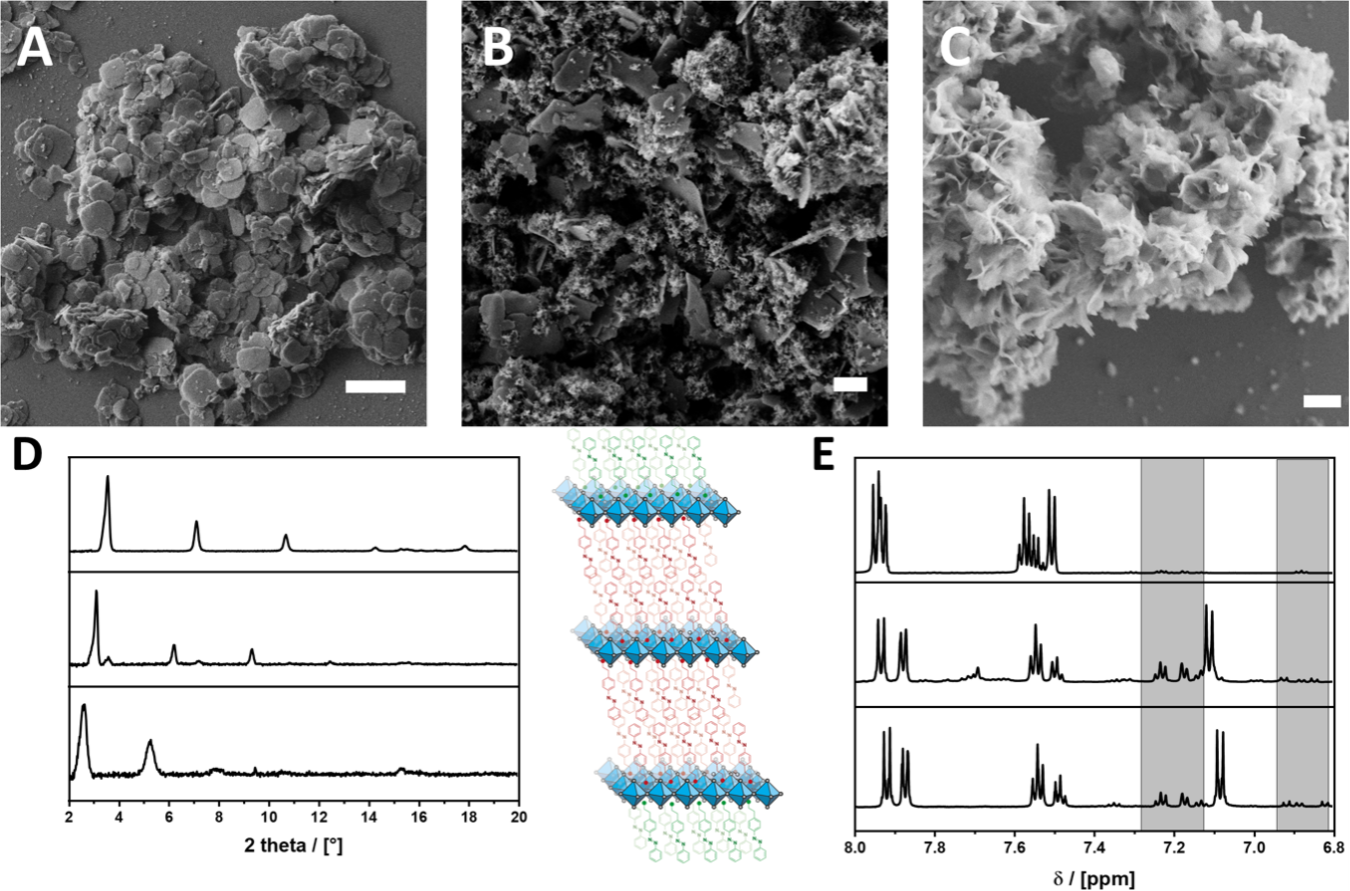
(A) SEM image of 2D-AzoC_2_, (B) SEM image of 2D-AzoOC_4_, (C) SEM image of 2D-AzoOC_12_, scale bar = 1 µm. (D) Powder X-ray diffraction (PXRD) pattern of the 2D LHPs with incorporated AzoC_2_, AzoOC_4_ and AzoOC_12_ with a layer thickness of *d*_(001)_ = 2.48 nm, *d*_(001)_ = 2.84 nm and *d*_(001)_ = 3.40 nm, respectively (from top to bottom). (E) ^1^H NMR spectra in the aromatic region from 8 to 6.8 ppm of 2D-AzoC_2_, 2D-AzoOC_4_ and 2D-AzoOC_12_ (from top to bottom), which were first dispersed in toluene, irradiated at 313 nm for 600 s, dried and dissolved in MeOD. Signals of the *cis*-isomers are highlighted in grey. The scheme shows the two types of azobenzene ligands in the 2D systems, i.e., molecules incorporated into the layers and species bound to the exterior surface of the particles.

2D LHPs, compared to their related 3D counterparts, consist only of one layer of PbBr_6_ octahedra with a large organic cation between the inorganic layers. The incorporation of a large organic cation is restricted by several factors. Primarily, the cation needs a coordinating headgroup that is able to ionically interact with the perovskite structure. In addition, the molecular projection along the *z*-axis should fit into the square defined by four corner-sharing octahedral [14]. Thus, the cross section of the ligand is a limiting factor, whereas there are (almost) no restrictions regarding the molecular length of the ligand. All of our synthesized azobenzene ligands with ammonium headgroup are able to coordinate to the perovskite network. Nevertheless, only AzoC_2_, AzoOC_4_ and AzoOC_12_ yield 2D LHPs. Considering the structure of AzoC_1_ the headgroup provides not enough space for the molecule to fit into the structure. The materials were examined by using a combination of methods to study their structure and electronic properties. In Figure 2D the powder X-ray diffraction (PXRD) patterns and the associated scanning electron microscopy (SEM) images of the obtained 2D LHPs with AzoC_2_, AzoOC_4_ and AzoOC_12_ are shown. For simplification the materials are named 2D-AzoX in the following.

All ligands yield a 2D LHP with a typical alignment of the PXRD reflexes. Sharp and defined reflexes obtained from 2D-AzoC_2_ and 2D-AzoOC_4_ imply the formation of an inorganic layer with only corner-sharing octahedra. The broadened reflexes of 2D-AzoOC_12_ suggest the formation of a crystal structure with corner- and face-sharing octahedra [43]. Both crystal types are direct semiconductors [44], which is why the structural differences are not important for our further studies. A plate-like appearance and the sharp reflexes of 2D-AzoC_2_ and 2D-AzoOC_4_ indicate highly ordered materials, which we explain by strong π-stacking forces between the organic ligands. 2D-AzoOC_12_ forms a more sponge-like unordered network and yields broader reflexes, which is due to the more flexible organic molecule.

**Figure 3:**
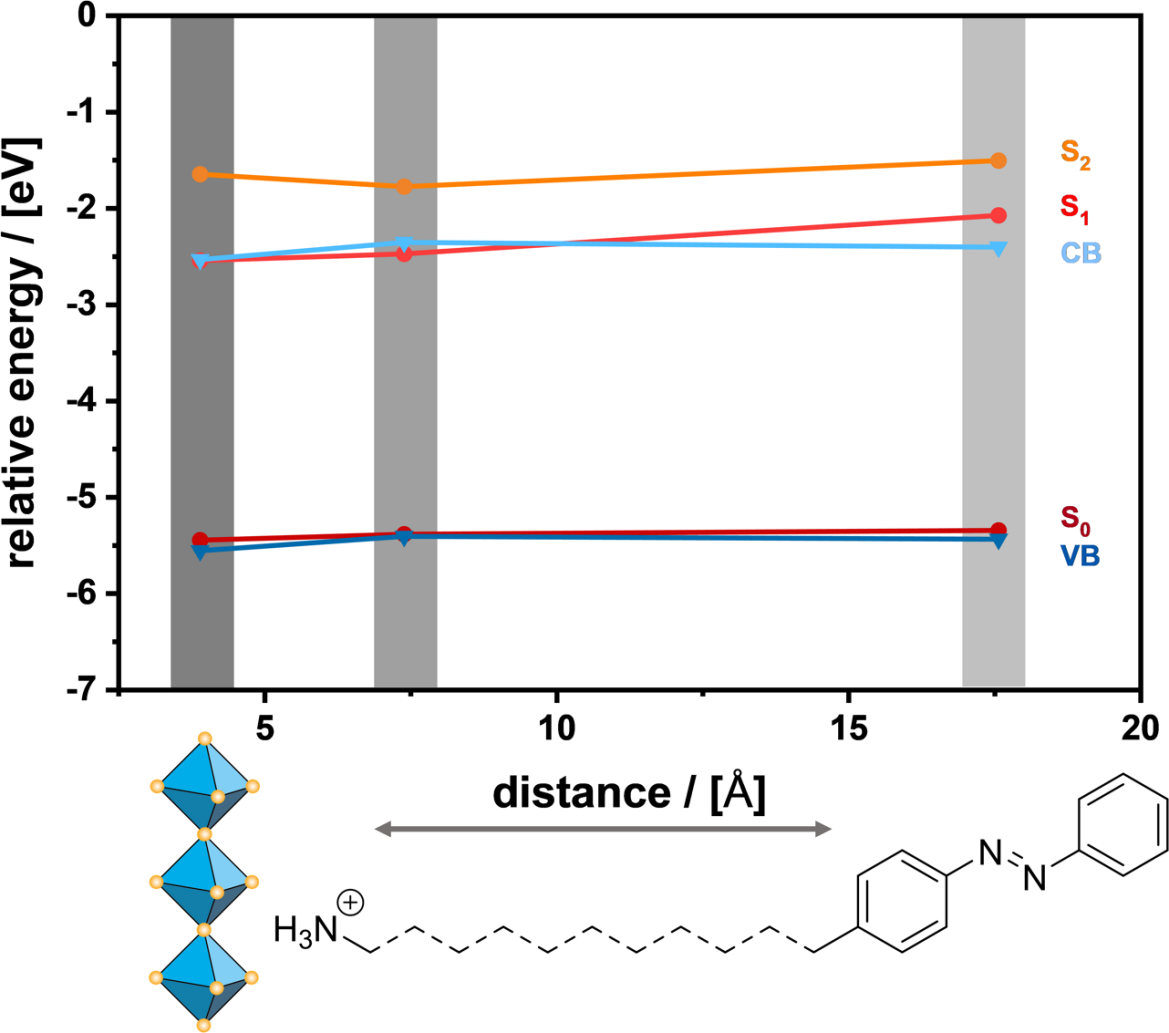
Energy-level diagram of 2D-AzoC_2_, 2D-AzoOC_4_ and 2D-AzoOC_12_ as function of the distance between the perovskite layer and the azobenzene moiety. UV–vis and PESA measurements can be found in Supporting Information File 1, Figure S15.

Our next goal is to check if photoswitching is still possible after integration into the perovskite. Sasai et al. reported the photoswitching of azobenzene molecules in a 2D LHP tracking the optical properties of the molecules [41]. The photoswitching properties of the integrated azobenzene molecules in our materials were determined using a combination of methods. A quantitative assertion can be made using ^1^H NMR spectroscopy of the dissolved particles, which gives a DOI. The conformational change is accompanied by a reduction of the molecule length, which is why a change in layer thickness should be observable in PXRD measurements. After irradiation of a dispersion of 2D-AzoC_2_, 2D-AzoOC_4_ and 2D-AzoOC_12_ at λ = 313 nm for 600 s, the materials were dissolved in MeOD and ^1^H NMR was measured (Figure 2E). According to UV–vis kinetic measurements of the dispersed materials a time period of 600 s is sufficient (see Supporting Information File 1, Figure S13). ^1^H NMR requires the dissolution of the materials in MeOD, which is indeed destructive. However, an exact DOI can be evaluated. After irradiation 2D-AzoC_2_ shows a very low DOI of 2.5%, while 2D-AzoOC_4_ and 2D-AzoOC_12_ show slightly increased values of 12.3% and 7.8%, respectively. Given the fact that the materials contain a very large amount of azobenzene ligands, the DOI is very low in all cases. Therefore, we think that only surface-coordinating azobenzene ligands are able to react under irradiation. A conformational change of the azobenzene moiety is accompanied with a decrease in length by 0.4 nm, which should result in smaller layer thicknesses [35]. If the isomerisation caused any change in structure or layer thickness this should be observed with PXRD. The required analysis is shown in Supporting Information File 1, Figure S14, for the particles after irradiation. Compared to the *d*_(001)_ values of the initial 2D materials, we could not observe any change. Considering the results of ^1^H NMR in combination with PXRD no significant photoswitching of the azobenzene molecules is detected. Recently published calculations showed that photoswitching of azobenzene on semiconducting TiO_2_ is suppressed due to a sudden oxidation of the chromophore. The formation of a heterogeneous charge-transfer complex disturbs the isomerisation and no photoswitching is observed [37]. To clarify possible electronical processes, the relative energies of valence band (VB) and conduction band (CB) were determined using solid-state UV–vis measurements in combination with PESA. Similar to the determination of the HOMO, the VB can be determined with PESA. Solid-state reflection spectra of the LHPs give information about the bandgap energy *E*_g_ of the semiconductor and therefore of the conduction band (CB). Relative energies for VBs and CBs, shown in Figure 3, are comparable for all 2D LHPs with integrated azobenzene ligands. Possible energy-transfer mechanisms are, thus, influenced exclusively by the distance between the inorganic layer and the azobenzene molecules. Regarding the energies, a charge transfer from CB to S_1_ is conceivable, likewise in the other direction.

The conclusions from our findings are: It is at least very questionable whether photoswitching of azobenzene species within the layers of the 2D hybrid perovskite phases takes place at all. The most likely explanation are steric factors. The photoisomerisation requires freedom to move for the molecular groups. The space within the layers is so limited that the activation barrier becomes too high and thus photoswitching cannot take place. Weaker π-stacking in 2D-AzoOC_4_ and 2D-AzoOC_12_ materials leads to more disordered morphologies, which explains the higher DOI of these materials. However, it should be considered that in the latter systems azobenzene molecules are not just present inside the layers of the particles, but also on the surface of the particles (Figure 2). The steric constraints for these surface-bound azobenzene molecules are much lower, and therefore photoswitching is much more likely. If no distinction is made between these two types of azobenzene species, the data relating to the photophysical properties of the material can easily be misinterpreted.

### 3D hybrid perovskite with azobenzene ligands on the surface

**Figure 4:**
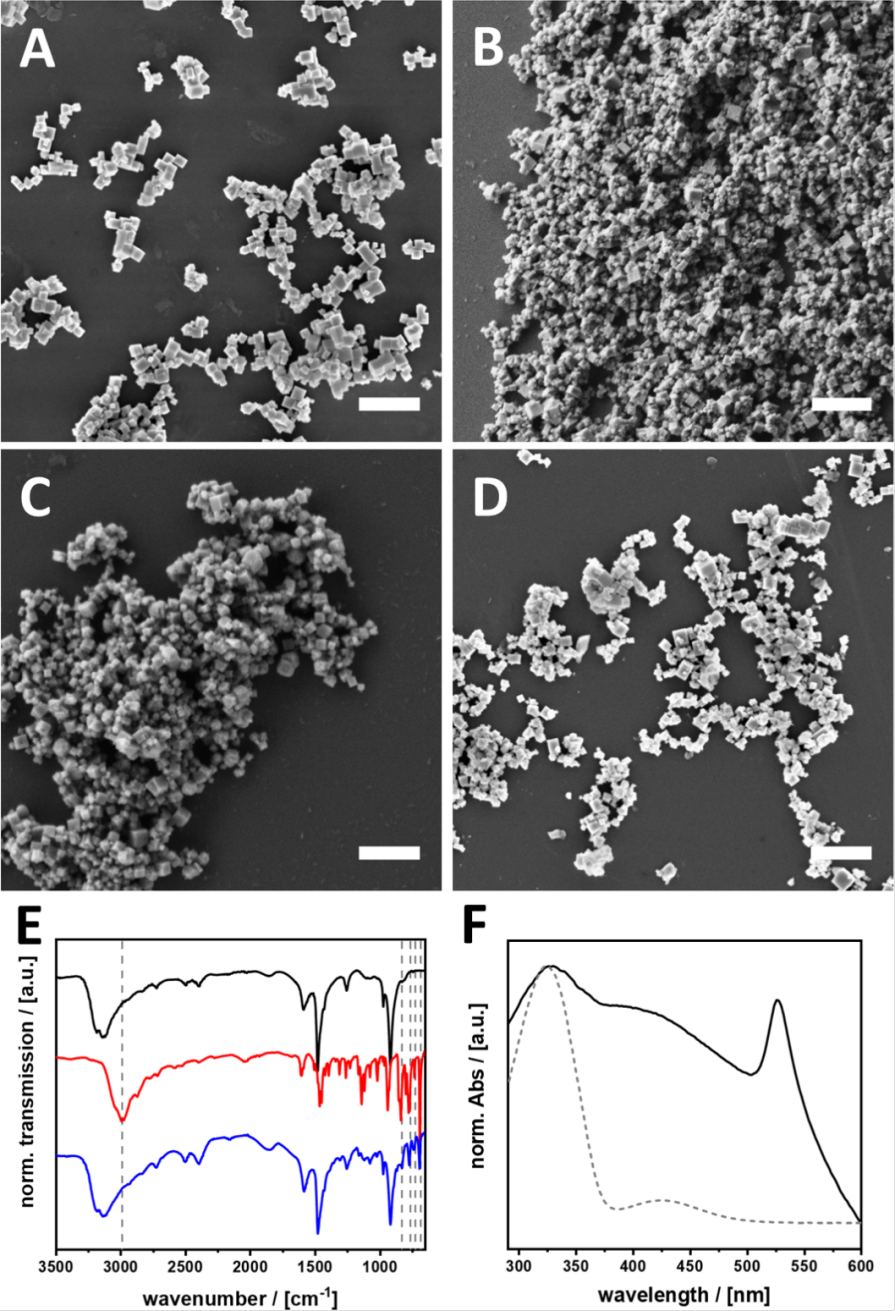
(A–D) SEM images of particles with (A) AzoC_1_, (B) AzoC_2_, (C) AzoOC_4_ and (D) AzoOC_12_ ligands on the surface, scale bar = 1 µm. (E) FTIR transmission spectrum of non-functionalized CH_3_NH_3_PbBr_3_ (black), AzoC_2_ (red) and 3D-AzoC_2_ (blue). Wave numbers at 2991, 832, 765, 727 and 685 cm^−1^ are highlighted in grey as significant vibrations of the azobenzene. (F) Normalized UV–vis absorption spectra of 3D-AzoC_2_ dispersed in toluene (black) and AzoC_2_ in H_2_O (grey, dashed).

For clarification of the issue mentioned in the previous section, we want to observe the photoswitching reaction of the ligands coordinated exclusively to the surface of 3D hybrid perovskite particles. With this approach it is possible to investigate only surface-dependent mechanisms. Because in the 3D systems, the azobenzene compounds are only attached to the surface, with much less steric hindrance compared to the sandwich situation in the 2D compounds. Thus, higher rates of photoswitching are expected. The ligands are more comparable with the surface-coordinated ligands in the 2D LHPs and only surface-dependent processes are investigated. To this end, we used the azobenzene compounds as capping agents during particle generation and avoided the formation of 2D structures. Furthermore, the physical investigations should be facilitated by increasing the surface-to-volume ratio of the perovskite particles, which is why we tried to prepare rather small crystals. The synthesis of the materials is described in detail in the Experimental part. Figure 4 shows SEM images of the resulting surface-functionalized particles. All particles have a cubic shape and their size varies from 100 to 300 nm. PXRD measurements prove that all particles exhibit the cubic perovskite structure, independent on the applied ligand (see also Supporting Information File 1, Figure S16). For simplification the particles are named 3D-AzoX in the following. The functionalization and presence of the azo compounds was verified using a combination of Fourier-transform infrared (FTIR) and UV–vis absorption spectroscopy. A characteristic vibration at 2991 cm^−1^, which can be associated to the N–H stretching vibrations of the ammonium headgroup (Figure 4E), vanishes completely for 3D-AzoC_2_ [45]. It is well known, that vibrations of surface-bound amines are quenched through coordination. Signals at 832, 765, 727 and 685 cm^−1^ can be assigned to the aromatic vibrations of the phenyl rings of azobenzene. These significant vibrations can as well be found in 3D-AzoC_2_ (blue), which indicates a successful functionalization.

The UV–vis absorption spectra, shown in Figure 4F, confirm these findings. 3D-AzoC_2_ shows absorption maxima at 518 and 324 nm. The absorption at 518 nm is assigned to the excitonic bandgap of the perovskite. The absorption at 324 nm indicates the coordination of the azobenzene ligand to the surface. For clarity only the spectra of 3D-AzoC_2_ are shown here, all other spectra can be found in Supporting Information File 1, Figure S17.

The steric constraints at the surface of the particles are expected to be much lower compared to the 2D systems described above. Whether azobenzene ligands attached to semiconductors can change conformation under irradiation with light is still a question that is being discussed. Rego et al. simulated a very fast charge transfer from azobenzene to the conduction band of TiO_2_, followed by a strong vibronic relaxation that excites the N–N stretching mode. As the excited state is directly quenched no S_2_→S_0_ transition takes place and the molecules remain in their conformation [37]. In contrast, Saeed et al. reported a completely reversible and improved *trans*–*cis* isomerisation of an azobenzene ligand coordinated to semiconducting CdTe quantum dots [38]. Possible mechanisms in our systems can be clarified by determination of the relative energies of MOs, VB and CB of the perovskite. The relative energies of the perovskite particles were determined using a combination of UV–vis direct reflection spectroscopy and PESA (see Supporting Information File 1, Figure S18). All relative energies can be found in Table 3. For the observation of the photoinduced isomerisation a combination of UV–vis absorption and ^1^H NMR was used. All particles were dispersed in toluene and irradiated for 600 s at 313 nm. The dried particles were dissolved in MeOD for the ^1^H NMR measurements.

**Table 3:** Relative energies of VB and CB, determined by using a combination of UV–vis direct reflection and PESA of all functionalized particles. All spectra can be found in Supporting Information File 1, Figure S18.

	AzoC_1_	AzoC_2_	AzoOC_4_	AzoOC_12_

valence band (VB)	−5.50 eV	−5.56 eV	−5.50 eV	−5.40 eV
conduction band (CB)	−3.20 eV	−3.27 eV	−3.18 eV	−3.10 eV
*E*_gap_ (UV–vis)	2.30 eV	2.29 eV	2.32 eV	2.30 eV

**Figure 5:**
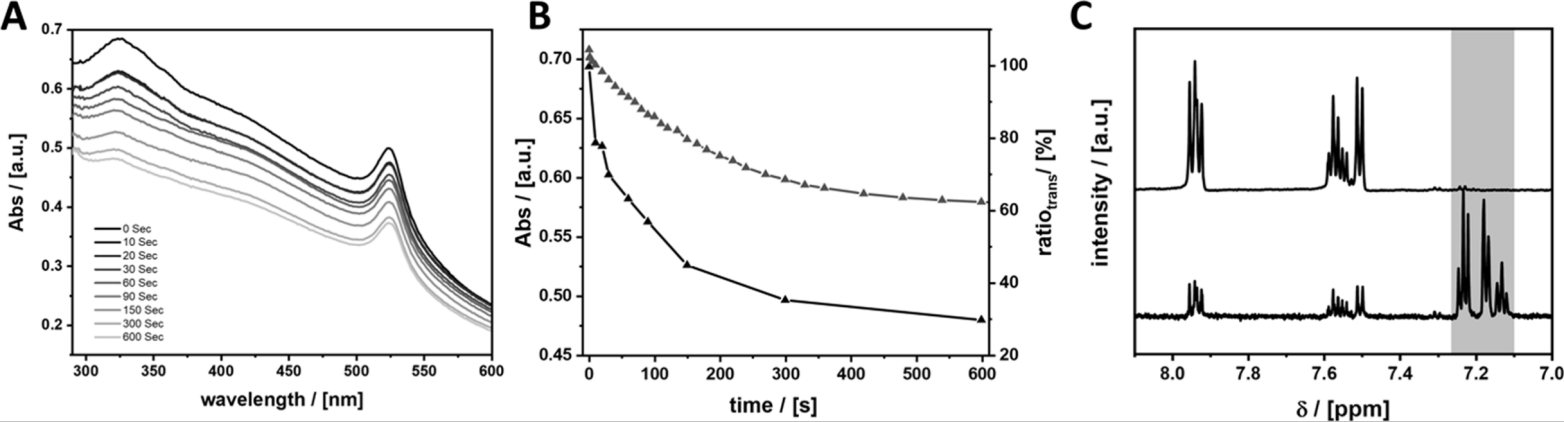
(A) UV–vis absorption kinetics of 3D-AzoC_2_ in toluene over a time period of 600 s. (B) Decrease of the absorption band at 325 nm of AzoC_2_ in H_2_O (grey) and 3D-AzoC_2_ (black), which is caused by the photoswitching reaction. (C) ^1^H NMR spectra of the dissolved 3D-AzoC_2_, top: before irradiation, bottom: after 600 s of irradiation at 313 nm. Signals of the *cis*-isomer are highlighted in light grey. It turns out that longer irradiation times are necessary for 3D-AzoOC_12_ to reach the photostationary state (see Supporting Information File 1, Figure S21H). It can be concluded, that the proximity to the perovskite improves the isomerisation under irradiation.

Irradiation at 313 nm of a dispersion of the particles in organic solution leads to the reduction of the absorption signal at 325 nm, which is expected for a successful photoisomerisation (Figure 5A). In addition, the spectra are shifted to lower intensities. This is caused by a partial destabilization of the particles and sedimentation, which can be seen by a general loss of absorption over the time (Figure 5B). Even without the conformational change of the surface-bound azo ligands, the particles are large enough to show slow sedimentation in Earth’s gravitational field. Irradiation, however, accelerates the process (see Supporting Information File 1, Figure S19). The conformational change is associated with a change of dipole moment from 0 D for the *trans*-isomer to 3 D for the *cis*-isomer [46]. Therefore, the polarity of the surface is reduced and particles tend to precipitate, which also known for other systems [47]. Because of this effect, the absorption of AzoC_2_ at 325 nm shows an exponential decay over the time of irradiation in relation to the excitonic bandgap at 524 nm. Thus, the drop in absorption at 325 nm signals indicates successful photoswitching.

For a quantitative analysis of the DOI, ^1^H NMR spectra of the dissolved particles were obtained. In Figure 5C, the ^1^H NMR spectra of the dissolved particles before (top) and after irradiation at 313 nm (bottom) are shown. Signals at 7.9 and 7.6–7.5 ppm belong to the *trans*-isomer and are less strong after 600 s of irradiation. Under irradiation, signals between 7.2 and 7.1 ppm appear, which can be assigned to the *cis*-isomer. Integration of the signals give a DOI of 71.9% for 3D-AzoC_2_. Compared to the DOI of approximately 39.4% of AzoC_2_ in MeOD after irradiation at 313 nm for over 4 h (see Supporting Information File 1, Figures S10–S12), the photoswitching reaction is strongly facilitated. The improvement can be observed for all ligands (Table 4). It can be concluded that the photoswitching from *trans*- to *cis*-isomer is enhanced through the coordination of the azobenzene to the semiconductor.

**Table 4:** DOI for dissolved functionalized 3D particles and free ligands in MeOD. ^1^H NMR spectra can be seen in the Supporting Information File 1, Figure S21.

	DOI of 3D particles	DOI of free ligand

AzoC_1_	89.0%	21.7%
AzoC_2_	71.9%	39.4%
AzoOC_4_	50.9%	25.1%
AzoOC_12_	79.9%	50.7%

Photoswitching as a reversible process can as well be observed for the *cis*–*trans* isomerisation. It can occur thermally, and indeed all azobenzene molecules return to the *trans*-conformation after equilibration [42]. The reverse reaction can also be triggered photochemically. Irradiation with blue light leads to a conformational change and therefore a rise in absorption of the S_0_→S_2_ transition (see Figure 1B). We investigated the reverse reaction by irradiating the particles at 438 nm after the irradiation at 313 nm (see Supporting Information File 1, Figure S20). Regarding the signal at 325 nm an exponential rise of the absorption is observed, indicating a successful photoisomerisation to the *trans*-conformation of the ligands on the perovskite surface. As already mentioned, the dispersion is destabilized through the preceding *trans*–*cis* isomerisation, which is not reversible. This behaviour is observed for all ligands. Therefore, an exact quantification of the photo-initiated back reaction is not possible, because precipitated particles could not be irradiated with blue light (438 nm) and this would falsify the determination of the DOI value.

The enhancement of the conformational change from *trans* to *cis* may originate from an energy transfer from the perovskite to the azobenzene. Here, the distance between the perovskite and the azobenzene moiety plays a crucial role. To verify the assertion of an energy transfer, further examination with time-dependent characterization techniques were quantified.

**Figure 6:**
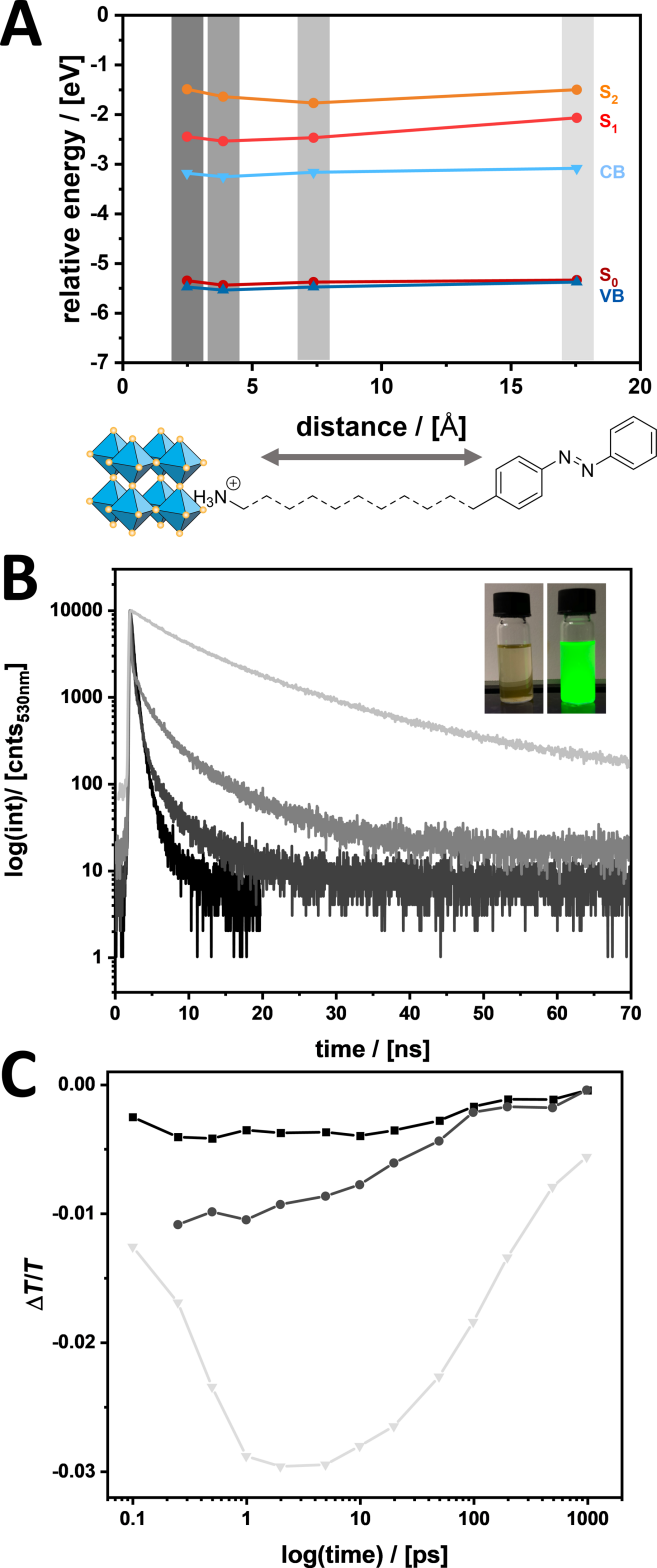
(A) Relative energy of MOs (S_0_, S_1_ and S_2_; see Table 2) of the azobenzene ligands and VB and CB of the perovskite particles in dependence on the distance between the perovskite and the azobenzene group. (B) Time-dependent PL measurements of a dispersion of 3D-AzoC_1_ (black), 3D-AzoC_2_ (dark grey), 3D-AzoOC_4_ (grey) and 3D-AzoOC_12_ (light grey). Excitation at λ_exc_ = 405 nm and recorded emission at λ_em_ = 530 nm. Top right: photography of 3D-AzoC_2_ dispersed in toluene (left) and alkyl-functionalized perovskite particles under illumination at 254 nm. (C) Decay of the ground state bleach obtained after an excitation pulse at 398 nm of transient absorption (TA) measurements for 3D-AzoC_1_ (at 522.5 nm, in black), 3D-AzoC_2_ (at 500 nm, in dark grey) and 3D-AzoOC_12_ (at 515 nm, in light grey), the original spectra can be found in Supporting Information File 1, Figure S23.

In a typical synthesis of nanoscaled hybrid perovskite particles with insulating alkyl ligands usually a bright luminescent material is obtained (see the photography in Figure 6B) [48–50]. Although the relative energies of VB and CB are in good agreement with literature, the visible photoluminescence (PL) of the particles with azobenzene ligands attached to the surface is completely quenched by a factor of 99.4%. We have investigated the PL signal of the remaining 0.6% for of all samples (see Supporting Information File 1, Figure S22), which shows a red-shift of the signal compared to the bandgap as expected.

The arrangement of the relative energies of MOs, VBs and CBs is similar for all azobenzene ligands used (Figure 6A). Energy transfer therefore depends only on the length of the spacer. The relative energies of VB and S_0_ ground state are equal. S_1_ and S_2_ are slightly above the CB. During irradiation at 313 nm, we propose a mechanism in which both components, perovskite and azobenzene, are excited. Photoswitching may be induced by two different effects: 1. Charge carriers in the excited azobenzene recombine under the classical conformation change from *trans*- to *cis*-isomer (S_1_→S_0_, n→π* transition). 2. Charge carriers from the excited perovskite are transferred to the azobenzene and induce a further switching. How is this possible, considering that the CB is below the S_1_ level? An energy of approximately 4.0 eV (313 nm) is more than sufficient for the perovskite to be excited. Electrons are lifted to a high virtual level. Usually trap states and lattice defects in the surface region lead to a high concentration of charge carriers and to radiative recombination with a bright luminescence [51–52]. Instead, electrons from the high virtual level can be transferred also to an excited state of the azobenzene. Therefore, decreased radiative recombination can be observed and increased photoswitching occurs.

Time-dependent fluorescence measurements reveal the distance-dependent energy transfer dynamics. We observe much shorter lifetimes for small insulating spacers (AzoC_1_ and AzoC_2_) than for longer spacers (AzoOC_4_ and AzoOC_12_), which can be seen in Figure 6B. As many other charge-carrier dynamics beside the radiative recombination, such as photorecycling and surface defects, affect the PL no (bi)exponential fit could be found [53–54]. Thus, the recorded measurements are discussed qualitatively. A small spacing between the perovskite surface and the azobenzene moiety provides a rapid energy transfer. A larger spacing leads to deceleration and the transfer is retarded through the insulating spacer. Therefore, radiative recombination is more likely. Our findings underline the proposed theory of an enhanced photoisomerisation through a direct connection of the azobenzene to the perovskite.

Finally, transient absorption spectroscopy (TAS) was applied. TAS is a versatile technique to observe rapid charge-carrier mechanisms in semiconductor–chromophore systems [55–56]. The observation of the excited bandgap of the perovskite is suitable to detect a distance dependence in our systems [57–58]. Dried particles were excited with a laser pulse of 398 nm. A ground-state bleach between 500 and 522.5 nm, which can be observed in all particle systems, originates from excited states in the bandgap (see Supporting Information File 1, Figure S23). Figure 6C shows the transient absorption of the ground-state bleach at 522.5 nm (3D-AzoC_1_), 500 nm (3D-AzoC_2_) and 515 nm (3D-AzoOC_12_) as a function of the time. Different mechanisms can be responsible for the interfacial recombination of charge carriers, such as trap states at the surface most probably from uncoordinated Pb^2+^ ions [59–60]. Here, instead of recombination, a charge-carrier transfer from a higher level to a LUMO of azobenzene is proposed. Also, the trend we observe is consistent with our findings for PL decays. A small spacing between the azobenzene moiety and the perovskite surface (AzoC_1_, AzoC_2_) leads to a rapid injection of excited electrons into the chromophore. A larger spacing (AzoOC_12_) suppresses the transfer and extended lifetimes are observed.

## Conclusion

**Figure 7:**
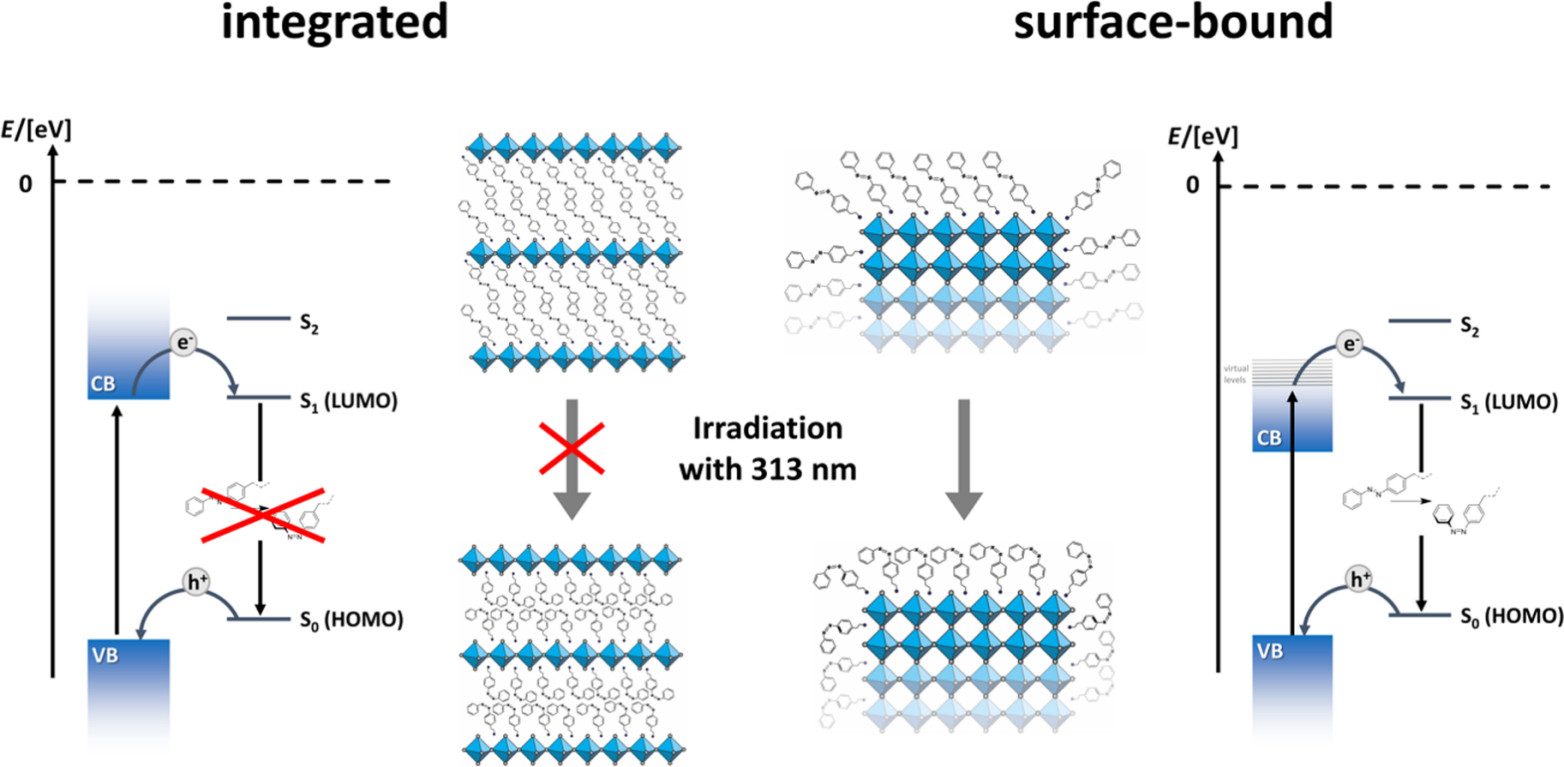
Comparison of the photoisomerisation and interfacial charge transfer processes for azobenzene incorporated into the hybrid perovskite phase (2D-system; left), and on the surface of the particles.

We have investigated the interfacial dynamics between 2D and 3D hybrid perovskite phases and novel photoswitching azobenzene ligands (R = AzoC_1_, AzoC_2_, AzoOC_4_ [41] and AzoOC_12_) coordinating to the surface as well as integrated into the material. Depending on their localization, the azobenzene constituents behave very differently. The conformational change from *trans*- to *cis*-isomer is suppressed when the ligands are incorporated into the 2D LHPs (Figure 7). However, a minor fraction of the azobenzene molecules is also present at the surface of the crystals. For a reliable analysis of the behaviour of these species, we have also studied 3D hybrid perovskite particles in detail. Findings from ^1^H NMR and UV–vis spectroscopy show that the isomerisation is even improved compared to the free ligands when they are coordinated to the external surface of the hybrid perovskite (Figure 7). We deduce that an energy transfer from a virtual level of the excited hybrid perovskite to the azobenzene occurs. Time-dependent PL and TA spectroscopy shed light onto the electronic mechanisms in the materials. Our findings indicate a distance dependency of the energy transfer. Small spacing provides a rapid energy transfer, whereas for larger spacing up to 17.6 Å for AzoOC_12_ a decelerated transfer is observed. Electronic transfers over long ranges are known for semiconductor–chromophore systems [57]. The spacing is therefore not long enough to cut off the interaction between the semiconductor and the azobenzene moiety.

Although the concentration of azobenzene is much higher for 2D LHPs, structural hindrance in the layers prohibits the photoswitching of the integrated molecules (Figure 7). Because, the relative energy levels are not changed depending on the positioning of the azobenzene, it cannot be excluded that charge transfer takes place in the 2D LHPs as well. However, this transfer definitely does not lead to conformational changes, opposite to azobenzene bound to the external surfaces.

## Experimental

### Chemicals

Lead(II) bromide (PbBr_2_, Sigma-Aldrich, 99.9% purity), methylamine (CH_3_NH_2_·H_2_O, Sigma-Aldrich, 33 wt % in ethanol), 4-aminobenzylamine (C_7_H_10_N_2_, Sigma-Aldrich, 99% purity) 4-(2-aminoethyl)aniline (C_8_H_12_N_2_, Sigma-Aldrich, 97% purity), nitrosobenzene (C_6_H_5_NO, Sigma-Aldrich, 97% purity), 9-fluorenylmethoxycarbonyl chloride (C_15_H_11_ClO_2_, Fmoc-Cl, Carbolution, 98% purity), piperidine (C_5_H_11_N, Sigma-Aldrich, 99.5% purity), hydrobromic acid (48 wt % in H_2_O, Sigma-Aldrich), 4-(phenylazo)phenol (C_12_H_10_N_2_O, Sigma-Aldrich, 98% purity), 1,4-dibromobutane (C_4_H_8_Br_2_, Sigma-Aldrich, 99% purity), 1,12-dibromododecane (C_12_H_24_Br_2_, Sigma-Aldrich, 98% purity), phthalimide potassium salt (C_8_H_4_KNO_2_, abcr, 98% purity) and hydrazine monohydrate (N_2_H_2_·H_2_O, acros organics, 99% purity) were used without further purification.

### Synthesis of azobenzene ligands

**Synthesis of AzoC_1_ and AzoC_2_:** 30 mmol 4-aminobenzylamine (*n* = 1) or 4-(2-aminoethyl)aniline (*n* = 2) was dissolved in 250 mL dichloromethane (DCM). After adding 36 mmol Fmoc-Cl, triethylamine (NEt_3_) was added dropwise under stirring. The suspension was stirred for 4 h at ambient temperature. The solvent was removed, and the residue was dissolved in ethyl acetate (EA) and methanol (MeOH) (5:1) and filtered over silica gel. The solvent was removed and a yellowish powder was obtained [61]. The product was characterized by using ^1^H NMR spectroscopy. ^1^H NMR (400 MHz, DMSO-*d*_6_) *n* = 1: δ 7.88 (d, 2H, CH, Fmoc), 7.69 (d, 2H, CH, Fmoc), 7.41 (t, 2H, CH, Fmoc), 7.32 (t, 3H, CH, Fmoc), 6.89 (d, 2H, CH, 2-(4-aminophenyl)methylamine), 6.50 (d, 2H, CH, 2-(4-aminophenyl)methylamine), 4.31 (d, 2H, CH_2_, Fmoc), 4.21 (t, 1H, CH, Fmoc), 4.00 (t, 2H, CH_2_); *n* = 2: δ 7.89 (d, 2H, CH, Fmoc), 7.69 (d, 2H, CH, Fmoc), 7.41 (t, 2H, CH, Fmoc), 7.33 (t, 2H, CH, Fmoc), 6.83 (d, 2H, CH, 2-(4-aminoethyl)aniline), 6.49 (d, 2H, CH, 2-(4-aminoethyl)aniline), 4.29 (d, 2H, CH_2_, Fmoc), 4.20 (t, 1H, CH, Fmoc) 3.11 (t, 2H, CH_2_), 2.52 (t, 2H, CH_2_).

The Fmoc-protected diamine was then added to a solution of nitrosobenzene (1.25 equiv) in glacial acetic acid. After stirring at 80 °C for 24 h a brown precipitate was obtained. The solid was dissolved in DCM and separated from the glacial acetic acid. After washing the organic phase with purified water three times the solvent was removed [62]. Chromatographic purification with EA and pentane (PE) (1:2) gave an orange solid. The product was characterized by using ^1^H NMR spectroscopy. ^1^H NMR (400 MHz, DMSO-*d*_6_) *n* = 1: δ 7.90–7.84 (m, 6H), 7.71 (d, 2H), 7.60 (d, 2H), 7.42 (t, 4H), 7.34 (m, 3H), 4.39 (d, 2H, CH_2_, Fmoc), 4.28 (t, 1H, CH, Fmoc), 4.03 (d, 2H, CH_2_); *n* = 2: δ 7.89–7.86 (m, 4H), 7.81 (d, 2H), 7.67–7.56 (m, 5H), 7.43–7.30 (m, 6H), 4.31 (d, 2H, CH_2_, Fmoc), 4.20 (t, 1H, CH, Fmoc), 3.28 (t, 2H, CH_2_), 2.83 (t, 2H, CH_2_).

For the deprotection of the amine the orange solid was dissolved in DCM. Under stirring piperidine was added dropwise. After 17 h at ambient temperature the solvent was removed and the product was purified chromatographically with EA/PE (1:1) with NEt_3_ (5.0%). The product was characterized by using ^1^H NMR. ^1^H NMR (400 MHz, DMSO-*d*_6_) *n* = 1: δ 7.90–7.82 (m, 4H), 7.63–7.51 (m, 5H), 3.82 (s, 2H, C-CH_2_-NH_2_); *n* = 2: δ 8.04 (s, 2H, NH_2_), 7.89-7.86 (m, 4H), 7.63-7.49 (m, 5H), 3.10 (m, 2H, CH_2_-CH_2_–NH_2_), 3.01 (t, 2H, CH_2_-CH_2_-C).

**Synthesis of AzoOC_4_** **and AzoOC_12_:** 60 mmol of 1,4-dibromobutane (*n* = 4) or 1,12-dibromododecane (*n* = 12) and 30 mmol 4-phenylazophenol were dissolved in 30 mL of aqueous NaOH (1 M) and heated under reflux overnight. After cooling a brown solid precipitated, which was filtered, dissolved in DCM and washed with purified water for three times. The organic phase was dried over MgSO_4_ and the solvent was removed. To remove excess dibromoalkane the solid was washed with *n*-hexane and dried under reduced pressure. The product was characterized by using ^1^H NMR. ^1^H NMR (400 MHz, DMSO-*d*_6_) *n* = 4: δ 7.90–7.83 (m, 4H, CN=CH-CH), 7.59–7.52 (m, 3H, CH-CH=CH), 7.13 (d, 2H, CH-CH=CO), 4.12 (t, 2H, O-CH_2_-CH_2_) 3.62 (t, 2H, CH_2_-CH_2_-Br), 1.99 (p, 2H, CH_2_-CH_2_-CH_2_), 1.88 (p, 2H, CH_2_-CH_2_-CH_2_); (400 MHz, CDCl_3_) *n* = 12: δ 7.93–7.87 (m, 4H, CN=CH-CH), 7.52–7.41 (m, 3H, CH-CH=CH), 7.00 (d, 2H, CH-CH=CO), 4.04 (t, 2H, O-CH_2_-CH_2_), 3.41 (t, 2H, CH_2_-CH_2_-Br), 1.89–1.79 (m, 4H), 1.42 (m, 2H), 1.29 (s, 14H).

The orange solid was dissolved in dimethylformamide (DMF) and potassium phthalimide (1.2 equiv) was added. Under stirring the solution was heated to 95 °C for 3 h. After cooling the solvent was removed and the obtained solid was dissolved in DCM, washed with purified water three times and dried over MgSO_4_. Removing the solvent gave an orange powder. For further processing the product with *n* = 12 was purified chromatographically from PE/EA (9:1) to pure EA. The product was characterized by using ^1^H NMR. ^1^H NMR (400 MHz, DMSO-*d*_6_) *n* = 4: δ 7.86–7.82 (8H, m), 7.58–7.49 (m, 3H, CH-CH=CH), 7.09 (d, 2H, CH-CH=CO), 4.10 (t, 2H, O-CH_2_-CH_2_), 3.65 (t, 2H, CH_2_-CH_2_-N), 1.81–1.76 (m, 4H, CH_2_-CH_2_-CH_2_); (500 MHz, CDCl_3_) *n* = 12: δ 7.92–7.87 (m, 8H), 7.51–7.42 (m, 3H, CH-CH=CH), 7.00 (d, 2H, CH-CH=CO), 4.04 (t, 2H, O-CH_2_-CH_2_), 3.40 (t, 2H, CH_2_-CH_2_-N), 1.88–1.82 (m, 4H), 1.48 (m, 2H), 1.29 (s, 14H).

To obtain the amine, the purified product was dissolved in tetrahydrofurane (THF)/ethanol (EtOH) (8:2) and hydrazine monohydrate (50.0 equiv) was added dropwise. The reaction was stirred at 80 °C under reflux for 3 h. After cooling a white precipitate was filtered and the remaining solvent was removed to yield an orange powder and excessive N_2_H_4_. The product was dissolved in DCM and washed with purified water three times. The organic phase was dried with MgSO_4_ and reduced. For *n* = 12 the product had to be chromatographically purified in EA/NEt_3_ (95:5) [41]. The product was characterized by using ^1^H NMR. ^1^H NMR (400 MHz, CDCl_3_) *n* = 4: δ 7.93–7.87 (m, 4H, CN=CH-CH), 7.52–7.41 (m, 3H, CH-CH=CH), 7.00 (d, 2H, CH-CH=CO), 4.06 (t, 2H, O-CH_2_-CH_2_), 2.79 (t, 2H, CH_2_-CH_2_-NH_2_), 1.87 (p, 2H, CH_2_-CH_2_-CH_2_), 1.65 (p, 2H, CH_2_-CH_2_-CH_2_); (400 MHz, CDCl_3_) *n* = 12: δ 7.91–7.86 (m, 4H, CN=CH-CH), 7.51–7.40 (m, 3H, CH-CH=CH), 7.00 (d, 2H, CH-CH=CO), 4.03 (t, 2H, O-CH_2_-CH_2_), 2.75 (t, 2H, CH_2_-CH_2_-NH_2_), 1.81 (m, 2H), 1.57–1.43 (m, 4H), 1.28 (s, 14H).

All amines were further dissolved in 1,4-dioxane and treated with HBr (1.5 equiv) to obtain the ammonium salt. The solvent was removed, the obtained brown solid was dissolved in sufficient EtOH and reprecipitated with diethyl ether (Et_2_O). The powder was filtrated, washed with Et_2_O and dried under reduced pressure. All products were characterized by using ^1^H NMR, ^13^C NMR and ESIMS (see Supporting Information File 1, Figures S1–S4) and used for the material synthesis. ^1^H NMR (400 MHz, DMSO-*d*_6_) *n* = 1: δ 7.90–7.82 (m, 4H, CN=CH-CH), 7.63–7.51 (m, 5H), 3.82 (s, 2H, C-CH_2_-NH_3_^+^); (400 MHz, DMSO-*d*_6_) *n* = 2: δ 7.90–7.82 (m, 4H, CN=CH-CH), 7.63–7.51 (m, 5H), 3.82 (s, 2H, C-CH_2_-NH_3_^+^); (400 MHz, DMSO-*d*_6_) *n* = 4: δ 7.90–7.83 (m, 4H, CN=CH-CH), 7.59–7.50 (m, 3H, CH-CH=CH), 7.14 (d, 2H, CH-CH=CO), 4.21 (t, 2H, O-CH_2_-CH_2_), 2.89 (m, 2H, CH_2_-CH_2_-NH_3_^+^), 1.84–1.71 (m, 4H, CH_2_-CH_2_-CH_2_); (400 MHz, DMSO-*d*_6_) *n* = 12: δ 7.89–7.83 (m, 4H, CN=CH-CH), 7.59–7.52 (m, 3H, CH-CH=CH), 7.12 (d, 2H, CH-CH=CO), 4.08 (t, 2H, O-CH_2_-CH_2_), 2.77 (m, 2H, CH_2_-CH_2_-NH_3_^+^), 1.75 (m, 2H), 1.52–1.43 (m, 4H), 1.25 (s, 14H).

The same procedure was used to produce the methylammonium bromide salt (CH_3_NH_3_Br, MABr).

### Synthesis of 2D perovskite phases

For the synthesis of a 2D perovskite phase a 1.0 M PbBr_2_ stock solution in DMF was prepared. 2.0 equiv of the appropriate Azo-(O)C*_n_* ligand was added to the solution. Under vigorous stirring 0.2 mL of the precursor solution was added quickly into 30 mL DCM (*n* = 2) or acetone (*n* = 4, 12). The suspension was stirred for 1 h, then the precipitation was centrifuged, washed three times with 3 mL DCM/acetone and dried under reduced pressure. The samples were kept under nitrogen atmosphere to prevent decomposition.

### Synthesis of functionalized 3D perovskite particles

For the material synthesis of 3D perovskite particles, a 1.0 M PbBr_2_ stock solution in dried triethylene glycol was prepared. For the preparation of the precursor MABr (0.9 equiv) and the appropriate Azo(O)C*_n_* ligand (0.1 equiv) were dissolved and the solution was cooled for at least 1 h. Under vigorous stirring 0.2 mL of the precursor solution was added quickly into 30 mL DCM. The suspension was stirred for 1 h, then the particles were centrifuged, washed three times with 3 mL DCM and then dried under reduced pressure. The samples were kept under nitrogen atmosphere to prevent decomposition.

### Characterization

X-ray diffraction (XRD) measurements of drop-cast particles on silicon substrates were obtained using an X-ray diffractometer (Bruker D8 Discover). Small-angle X-ray scattering (SAXS) patterns were obtained using a Bruker Nanostar. Scanning electron microscopy (SEM) images of drop-cast particles on silicon substrates were obtained using a Zeiss Auriga Crossbeam and a Zeiss Gemini Crossbeam microscope. UV–vis measurements of drop-cast particles on a glass substrate were acquired with an Agilent 8453 Carry 5000 spectrometer with an integrating sphere. For evaluation of the bandgap the Kubelka–Munk method was used. UV–vis measurements of particles in suspension were obtained with a Varian Cary 100 Scan device. UV–vis kinetic measurements were acquired with a Varian Cary 50 spectrometer. Photon electron spectroscopy (on air; PESA) of drop-cast particles on a glass substrate were acquired with a Riken Keiki AC-2 photoelectron spectrometer. Stationary as well as time-resolved photoluminescence (PL) measurements of particles suspended in toluene were obtained using a FluoTime 300 from Picoquant spectrometer. Transient absorption spectra (TAS) were measured of drop-cast particles on glass substrates. NMR measurements of particles were carried out in a Bruker Avance III 600 device.

## Supporting Information

NMR, ESIMS, UV–vis (kinetic) and PESA measurements, and DFT calculations of the free ligands; UV–vis (kinetic, direct reflection), PESA, PXRD and ^1^H NMR measurements of the (dissolved) 2D-LHPs. PXRD, IR, UV–vis (absorption in dispersion, direct reflection, kinetic), PESA, PL, TAS and ^1^H NMR measurements of the (dissolved) 3D hybrid perovskite particles.

File 1 Additional experimental data.
