# Supplementary material for: Interfacial charge transfer processes in 2D and 3D semiconducting hybrid perovskites: azobenzene as photoswitchable ligand
Source: Beilstein J Nanotechnol. 2020 Mar 17;11:466–79. doi: 10.3762/bjnano.11.38 (PMC7113553; doi:10.3762/bjnano.11.38)
Supplement: File 1 — Additional experimental data. [file Beilstein_J_Nanotechnol-11-466-s001.pdf]

BEILSTEIN JOURNAL OF NANOTECHNOLOGY

Supporting Information

for

Interfacial charge transfer processes in 2D and 3D semiconducting hybrid perovskites: azobenzene as photoswitchable ligand

Nicole Fillafer, Tobias Seewald, Lukas Schmidt-Mende and Sebastian Polarz

Beilstein J. Nanotechnol. **2020,** *11,* 466–479. doi:10.3762/bjnano.11.38

Additional experimental data

License and Terms: This is a supporting information file under the terms of the Creative Commons Attribution License (http://creativecommons.org/licenses/by/4.0). Please note that the reuse, redistribution and reproduction in particular requires that the authors and source are credited.
The license is subject to the *Beilstein Journal of Nanotechnology* terms and conditions: (https://www.beilstein-journals.org/bjnano)

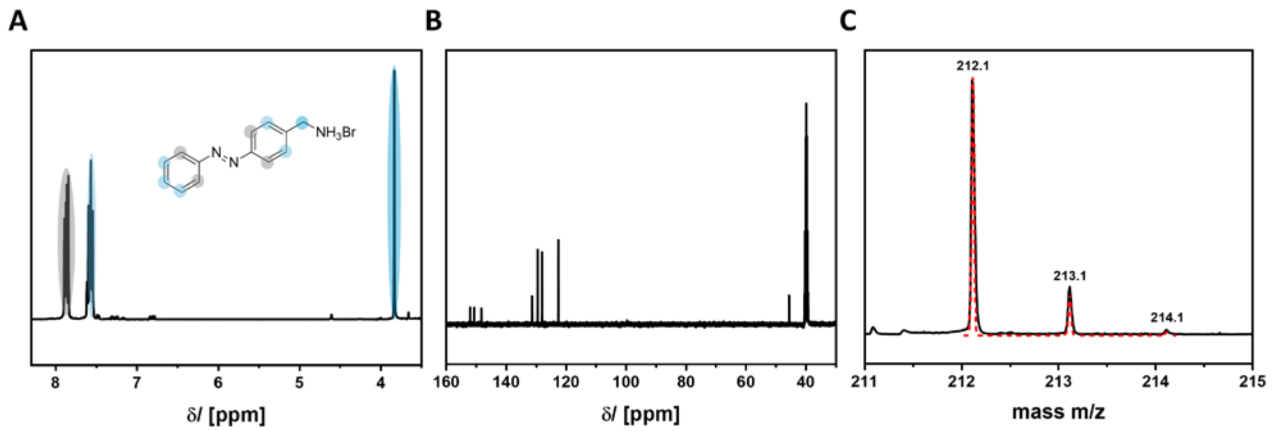

Figure S1: (A) ^{1}H NMR spectrum of AzoC$_1$ in DMSO-d_6, H atoms are marked. (B) ^{13}C NMR spectrum of AzoC$_1$ in DMSO-d_6, (C) ESI-MS spectrum of AzoC$_1$, black line: experimental, red dashed line: calculated.

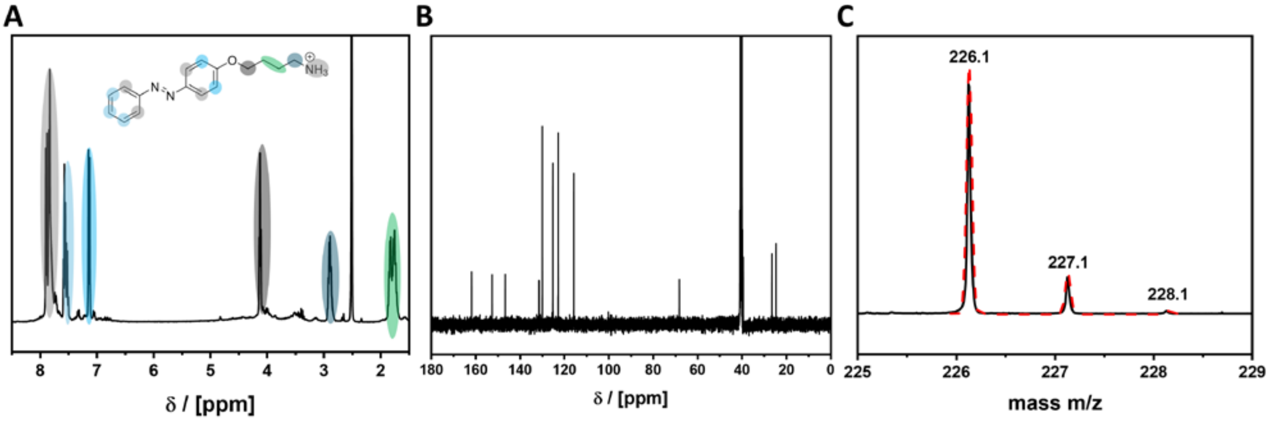

Figure S2: (A) ^{1}H NMR spectrum of $AzoC_2$ in DMSO-d_6, H atoms are marked. (B) ^{13}C NMR spectrum of $AzoC_2$ in DMSO-d_6, (C) ESI-MS spectrum of $AzoC_2$, black line: experimental, red dashed line: calculated.

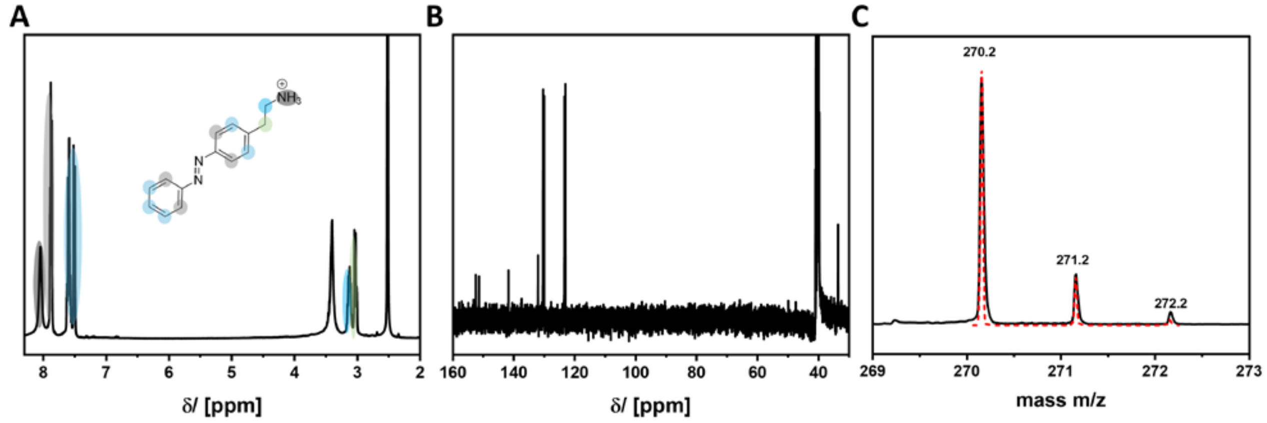

Figure S3: (A) ^{1}H NMR spectrum of AzoOC4 in DMSO-d_6, H atoms are marked. (B) ^{13}C NMR spectrum of AzoOC4 in DMSO-d_6, (C) ESI-MS spectrum of AzoOC4, black line: experimental, red dashed line: calculated.

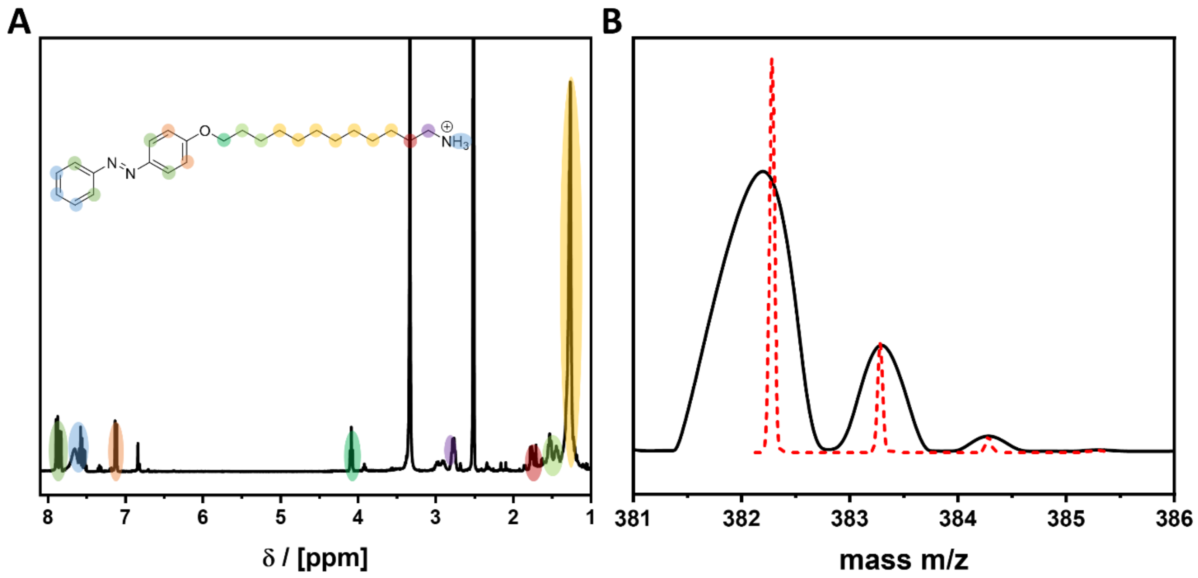

Figure S4: (A) ^{1}H NMR spectrum of AzoOC12 in DMSO-d_6, H atoms are marked. ^{13}C NMR was measured but the concentration was too low. (B) ESI-MS spectrum of AzoOC12, black line: experimental, red dashed line: calculated.

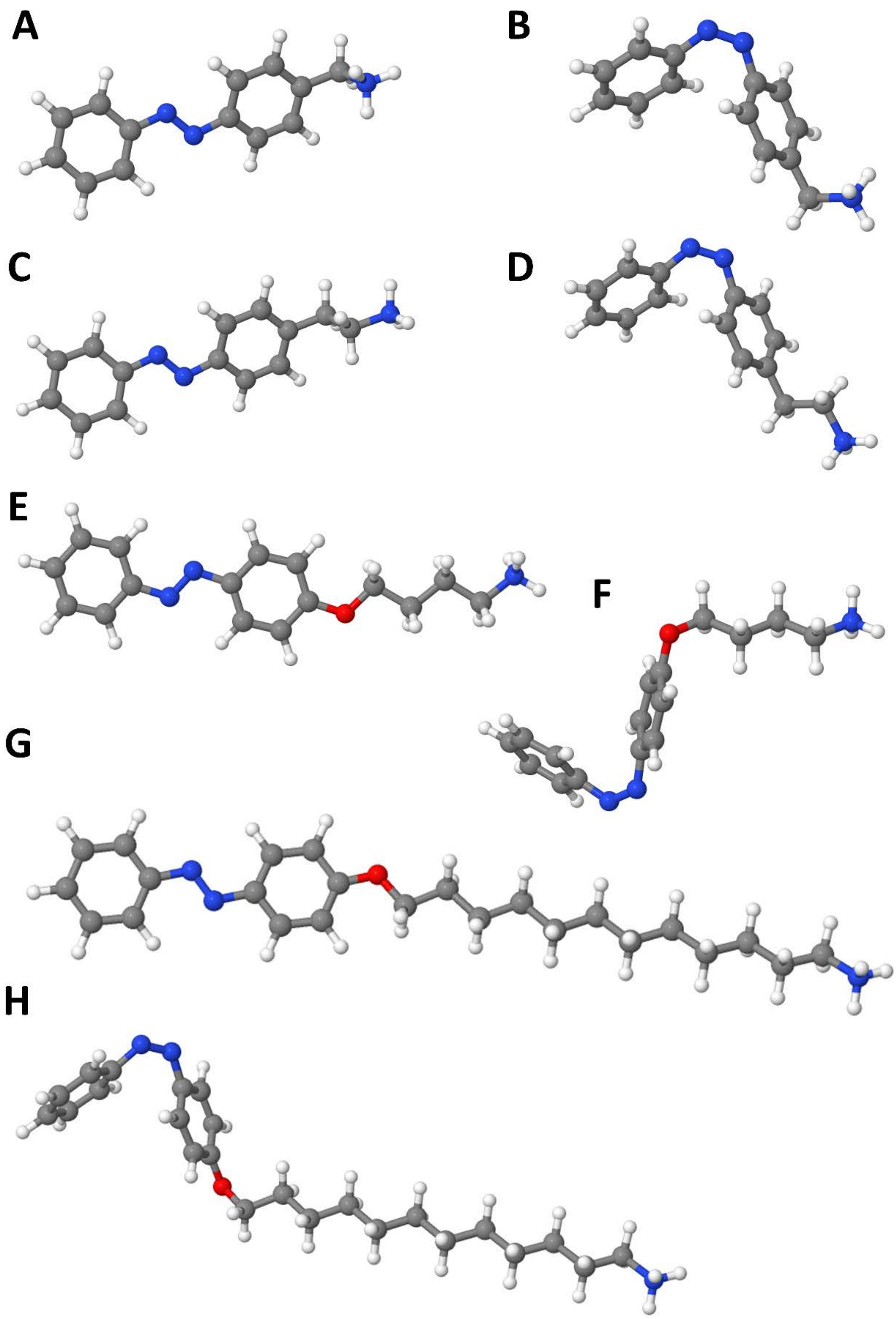

Figure S5: (A, B) DFT calculated structure of *trans*-AzoC$_1$ and *cis*-AzoC$_1$, (C, D) DFT-calculated structure of *trans*-AzoC$_2$ and *cis*-AzoC$_2$, (E, F) DFT calculated structure of *trans*-AzoOC$_4$ and *cis*-AzoOC$_4$, (G, H) DFT calculated structure of *trans*-AzoOC$_{12}$ and *cis*-AzoOC$_{12}$, all structures were created and measured with Jmol.

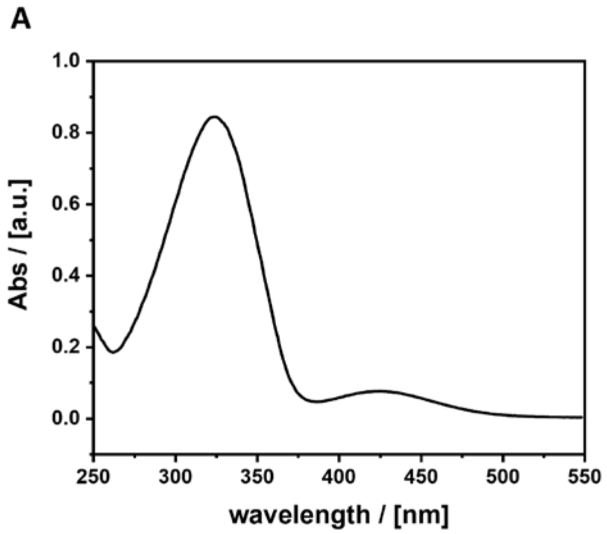

Figure S6: (A) UV–vis spectrum of $AzoC_1$ in H_2O, (B) PESA spectrum of $AzoC_1$.

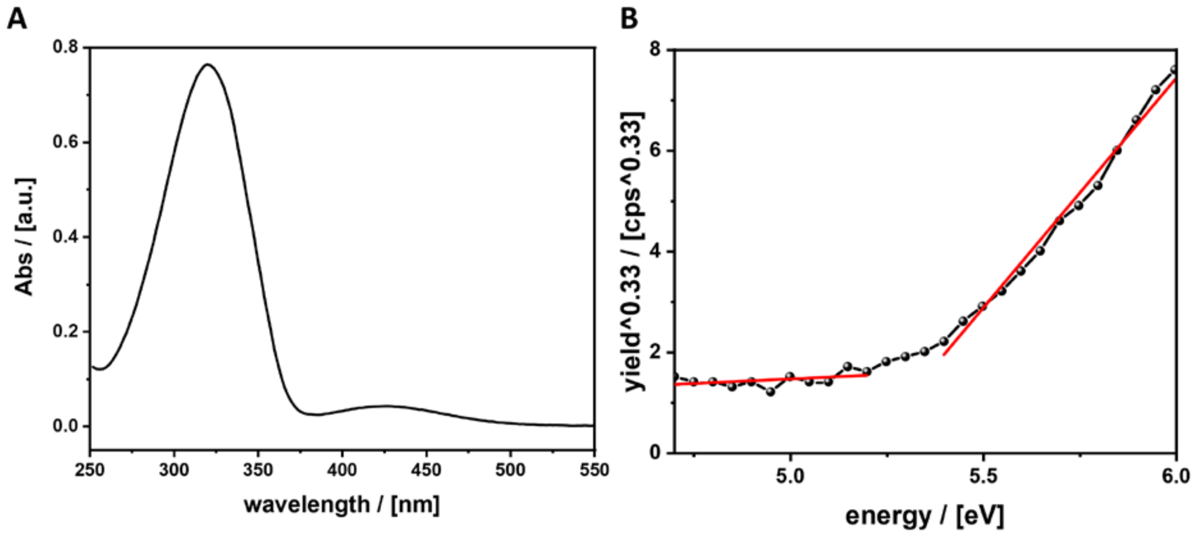

Figure S7: (A) UV–vis spectrum of AzoC$_2$ in H_2O, (B) PESA spectrum of AzoC$_2$.

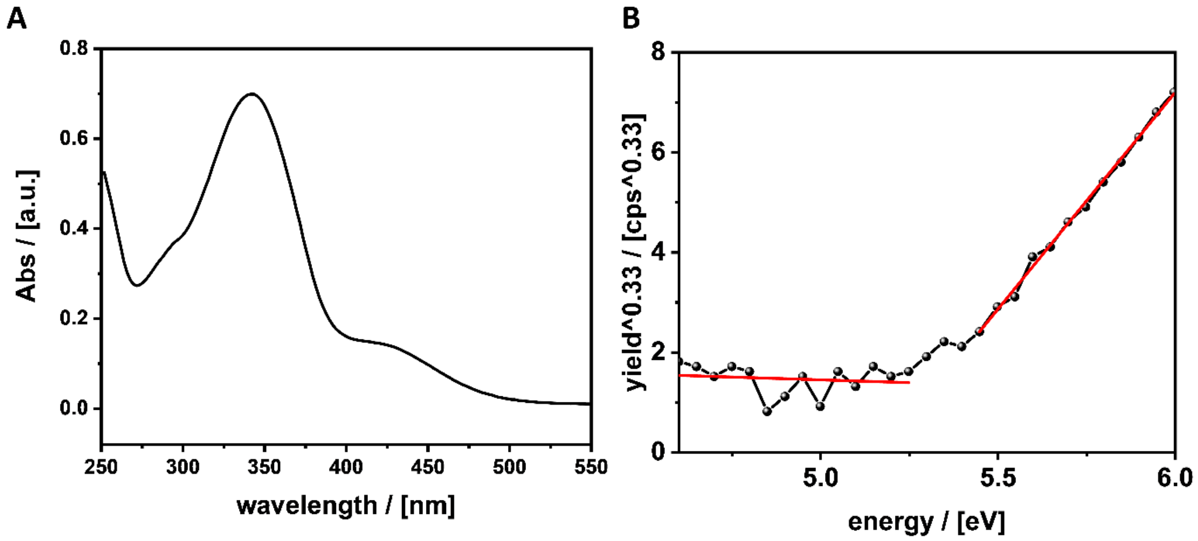

Figure S8: (A) UV–vis spectrum of $AzoOC_4$ in H_2O, (B) PESA spectrum of $AzoOC_4$.

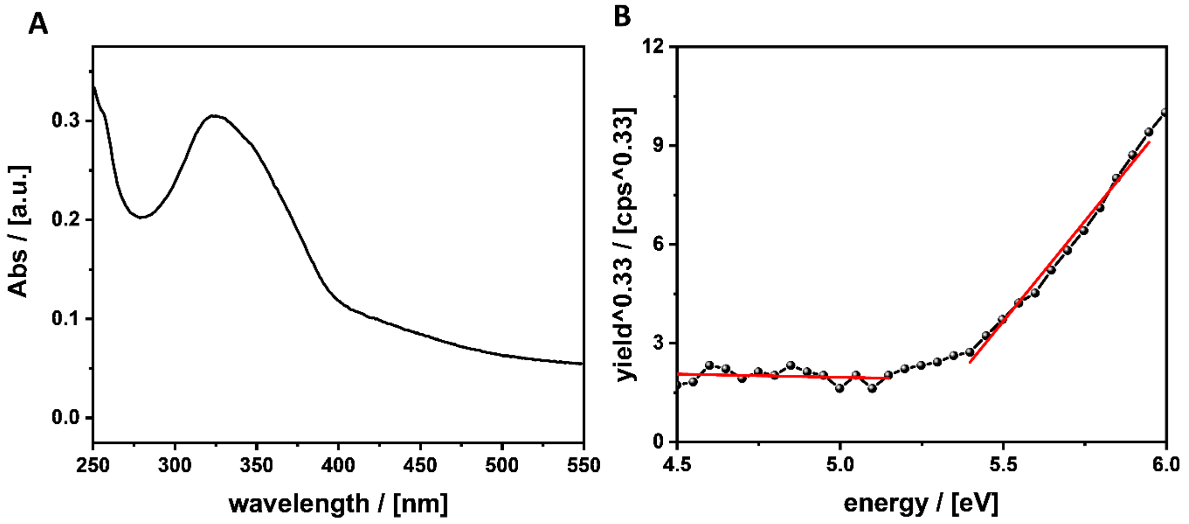

Figure S9: (A) UV–vis spectrum of $AzoOC_{12}$ in H_2O, (B) PESA spectrum of $AzoOC_{12}$.

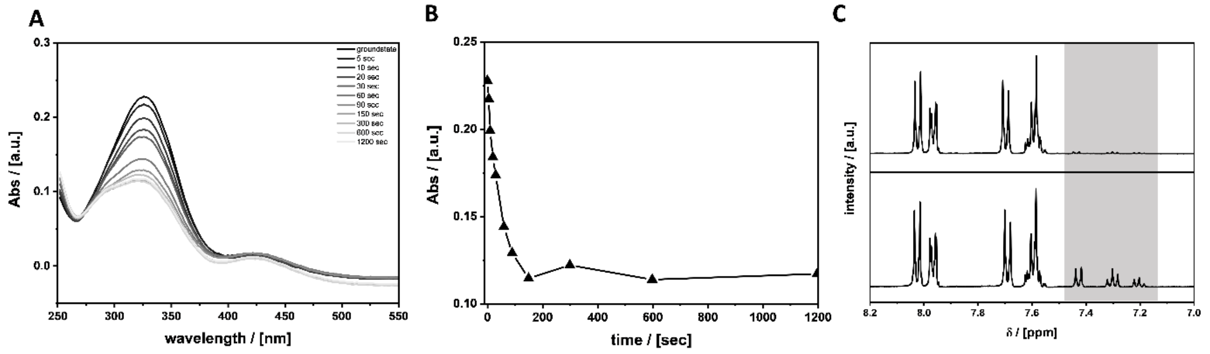

Figure S10: (A) UV–vis kinetic measurement of $AzoC_1$ in purified H_2O. The solution was irradiated at 313 nm, black: beginning of the reaction, light grey: ending of the reaction (B) absorption at 326 nm over a time period of 1200 s, (C) ^1H NMR of $AzoC_1$ in MeOD from 8.0 to 7.2 ppm, before (top) and after irradiation (bottom) at 313 nm for 1 h. Highlighted in grey is the signal of the *cis*-isomer. The DOI is 21.7%.

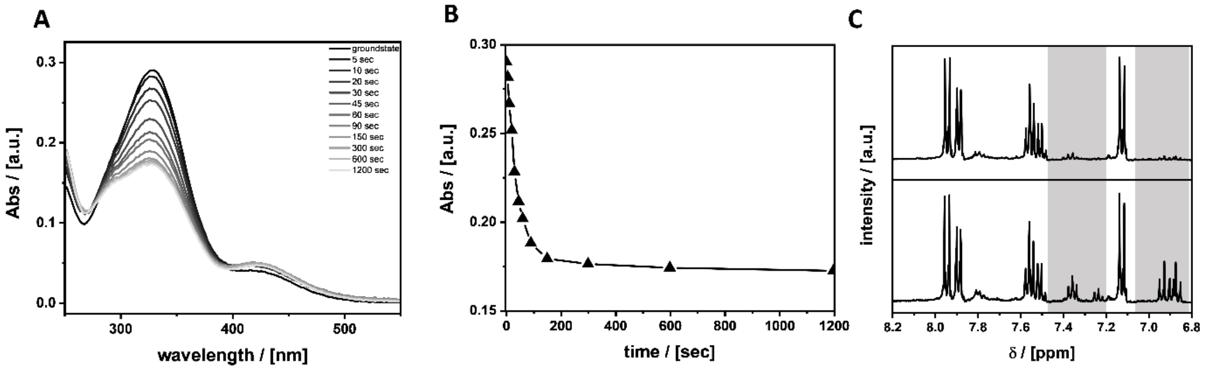

Figure S11: (A) UV–vis kinetic measurement of AzoOC$_4$ in purified H_2O. The solution was irradiated at 313 nm, black: beginning of the reaction, light grey: ending of the reaction (B) absorption at 328 nm over a time period of 1200 s. (C) ^{1}H NMR of AzoC$_1$ in MeOD from 8.0 to 7.2 ppm, before (top) and after irradiation (bottom) at 313 nm for 1 h. Highlighted in grey is the signal of the *cis*-isomer. The DOI is 25.1%.

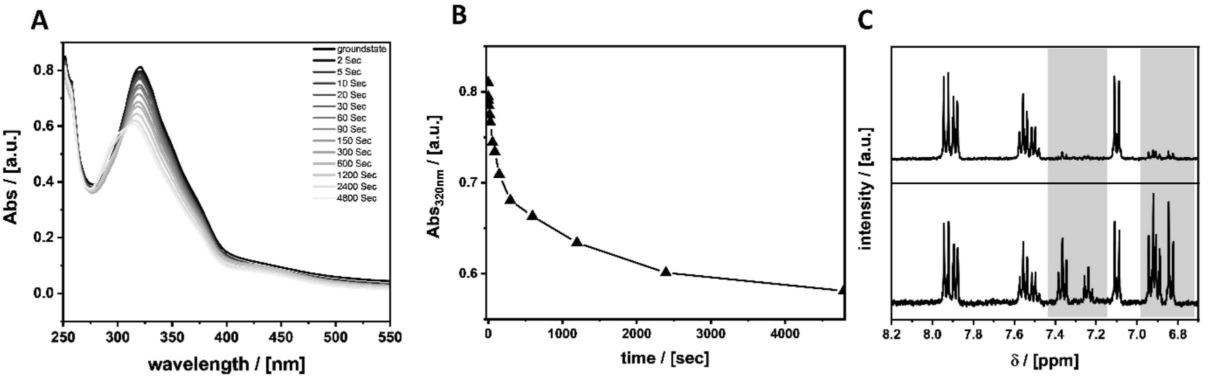

Figure S12: (A) UV–vis kinetic measurement of AzoOC$_{12}$ in purified H_2O. The solution was irradiated with 313 nm, black: beginning of the reaction, light grey: ending of the reaction (B) absorption at 328 nm over a time period of 4800 s. (C) ^{1}H NMR of AzoC$_{12}$ in MeOD from 8.0 to 7.2 ppm, before (top) and after irradiation (bottom) at 313 nm for 1 h. Highlighted in grey is the signal of the *cis*-isomer. The DOI is 50.7%.

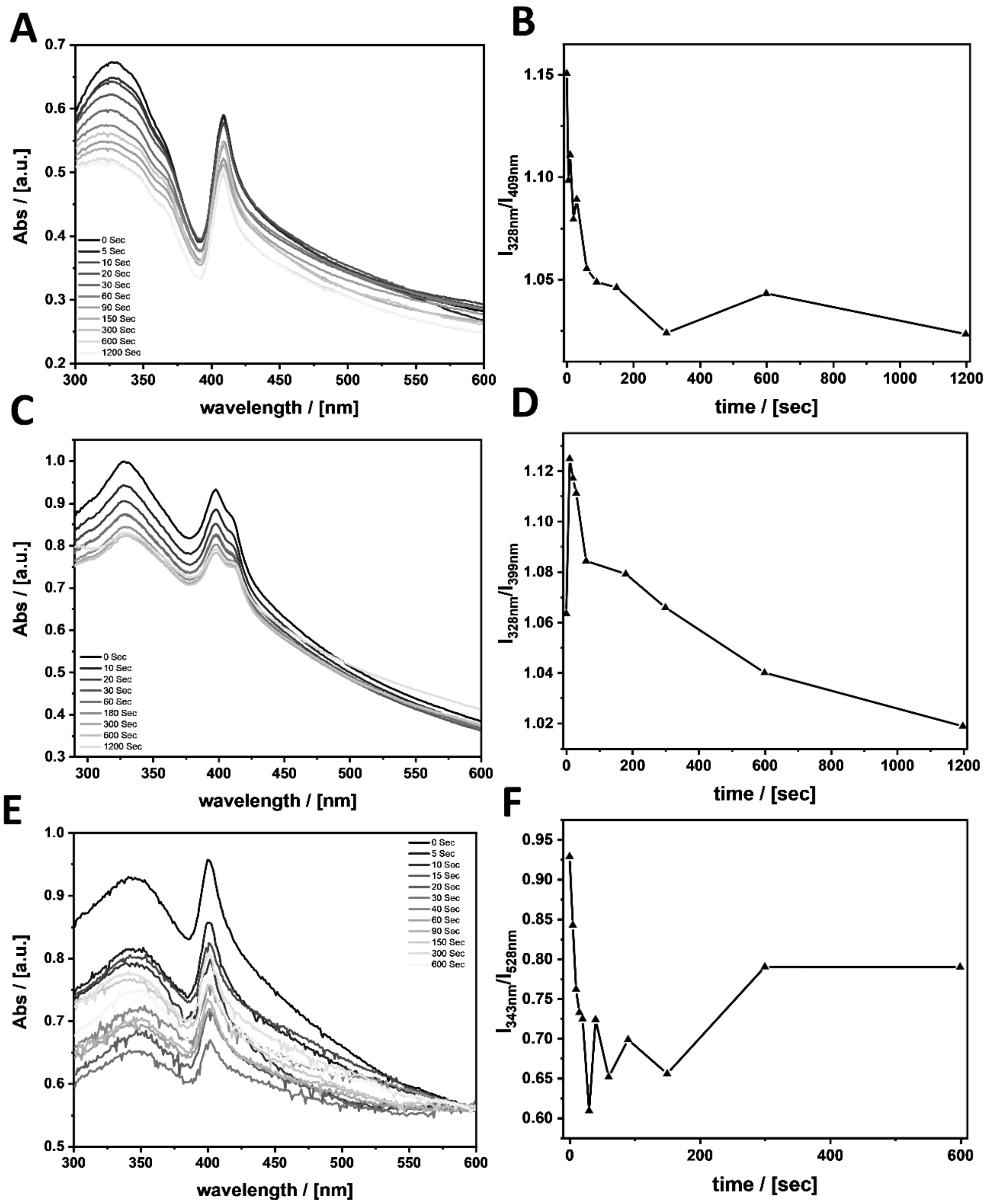

Figure S13: (A, C, E) UV–Vis measurements of dispersions of 2D-AzoC$_2$, 2D-AzoOC$_4$ and 2D-AzoOC$_{12}$ in toluene during irradiation at 313 nm. (B, D, F) Intensity of absorbance at the absorption maximum of the chromophore divided by the intensity of the absorbance of the excitonic bandgap signal. As the dispersions were not stable they were referenced to the signal of the bandgap.

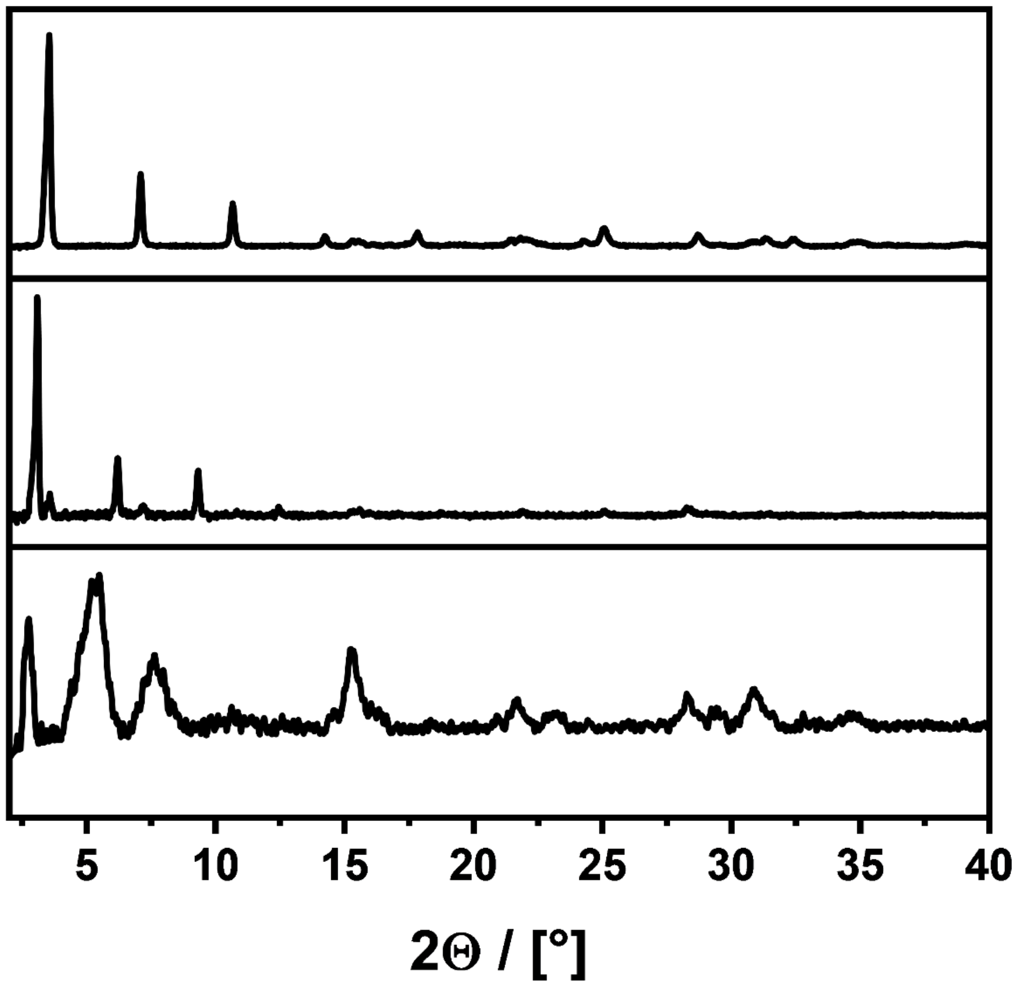

Figure S14: PXRD patterns of 2D-AzoC$_2$, 2D-AzoOC$_4$ and 2D-AzoOC$_{12}$ (top to bottom) after irradiation at 313 nm for 20 min. A layer spacing of $d_{(001)}$ = 2.48 nm, $d_{(001)}$ = 2.84 nm and $d_{(001)}$ = 3.40 nm, respectively, from top to bottom was found. No change compared to Figure 2A in the main manuscript can be found.

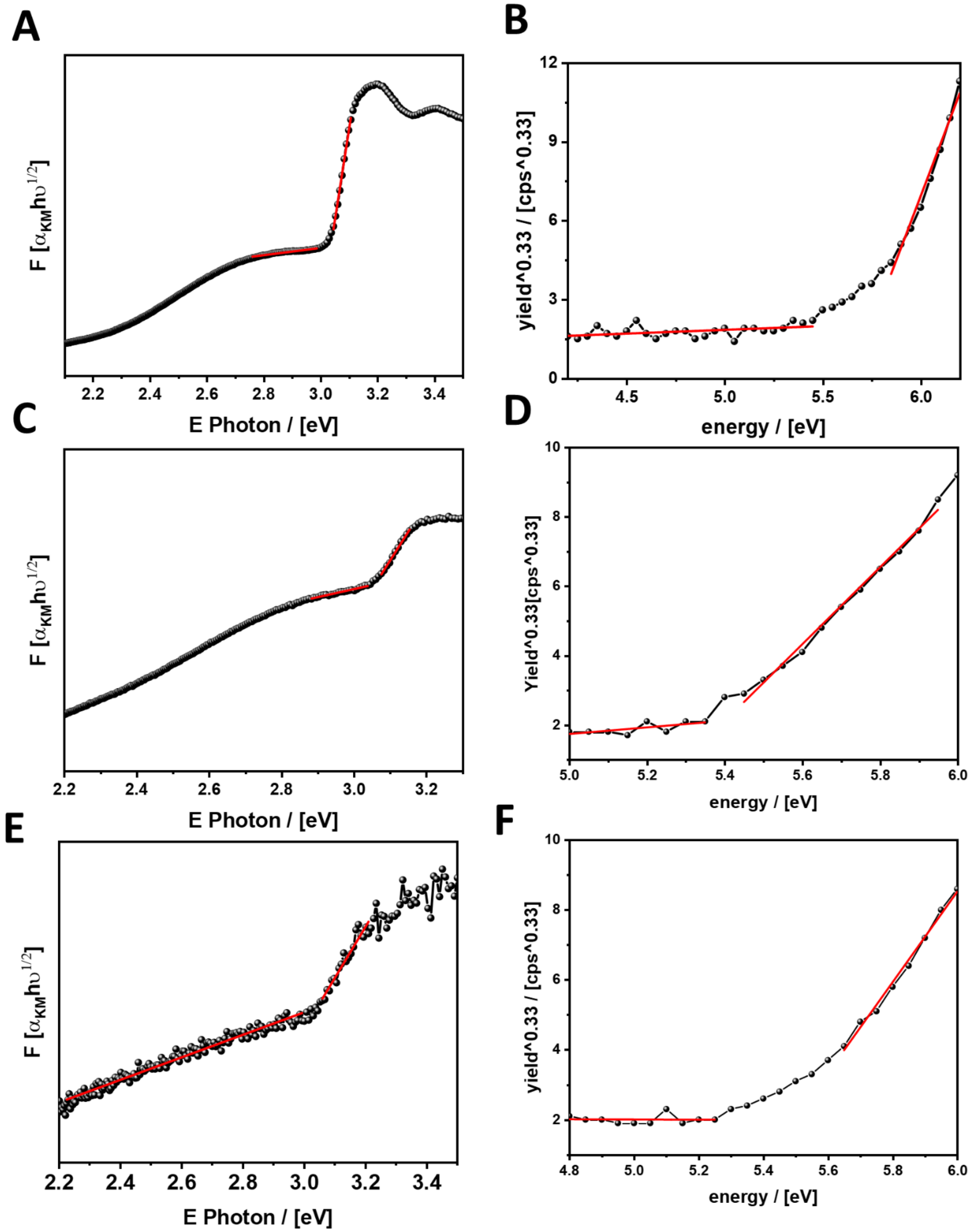

Figure S15: (A, B) Kubelka–Munk evaluation and PESA of 2D-AzoC_2, (C, D) Kubelka–Munk evaluation and PESA of 2D-AzoOC_4. (E, F) Kubelka–Munk evaluation and PESA of 2D-AzoOC_{12}. The intersection of baseline fit and slope fit gives the bandgap energy E_g or the relative energy E_{VB} of the valence band, respectively.

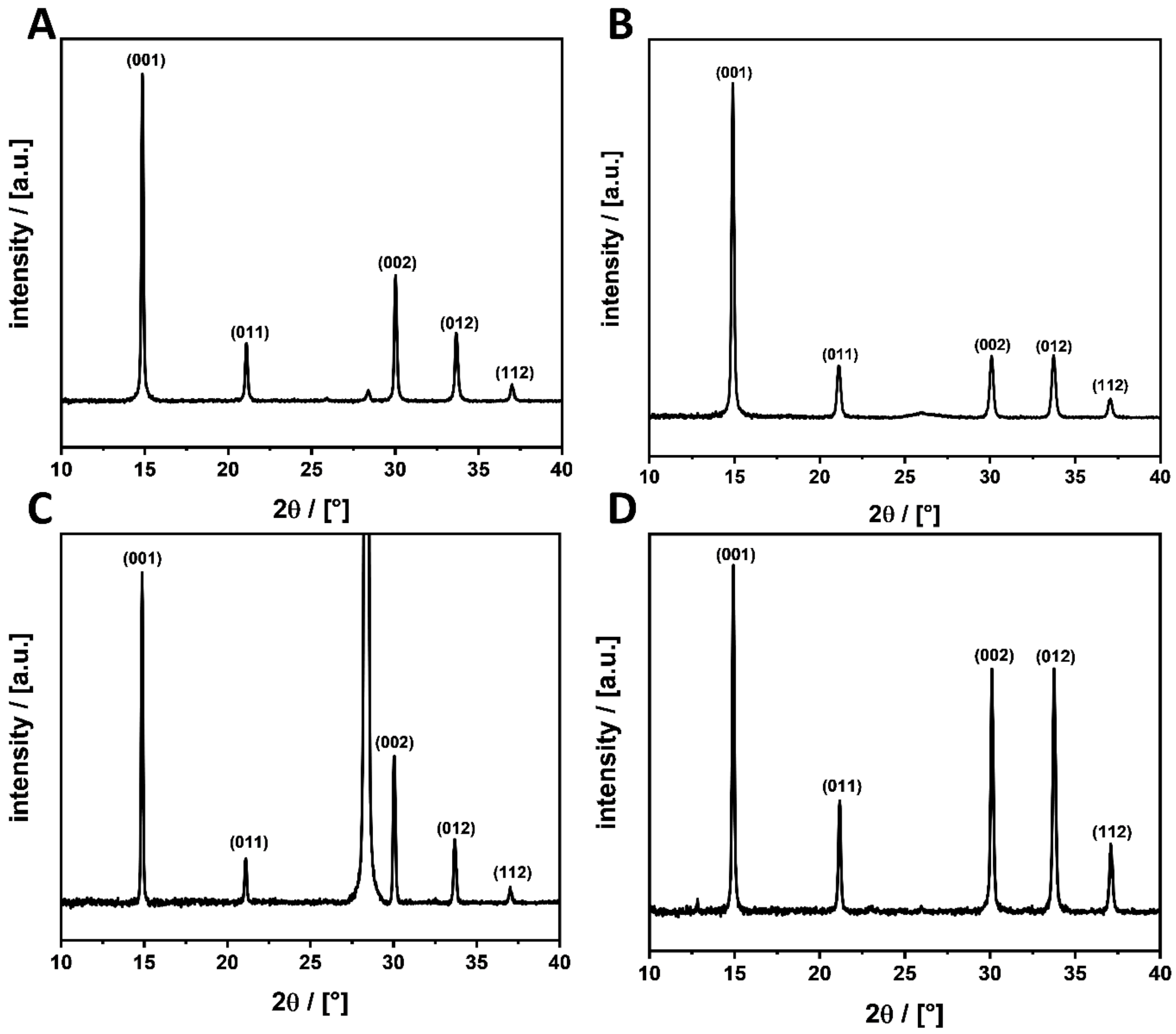

Figure S16: (A) PXRD pattern of 3D-AzoC$_1$, (B) PXRD pattern of 3D-AzoC$_2$, (C) PXRD pattern of 3D-AzoOC$_4$, (D) PXRD pattern of 3D-AzoOC$_{12}$. All reflexes originate from $CH_3NH_3PbBr_3$, the reflex at 2θ = 28° originates from the Si substrate.

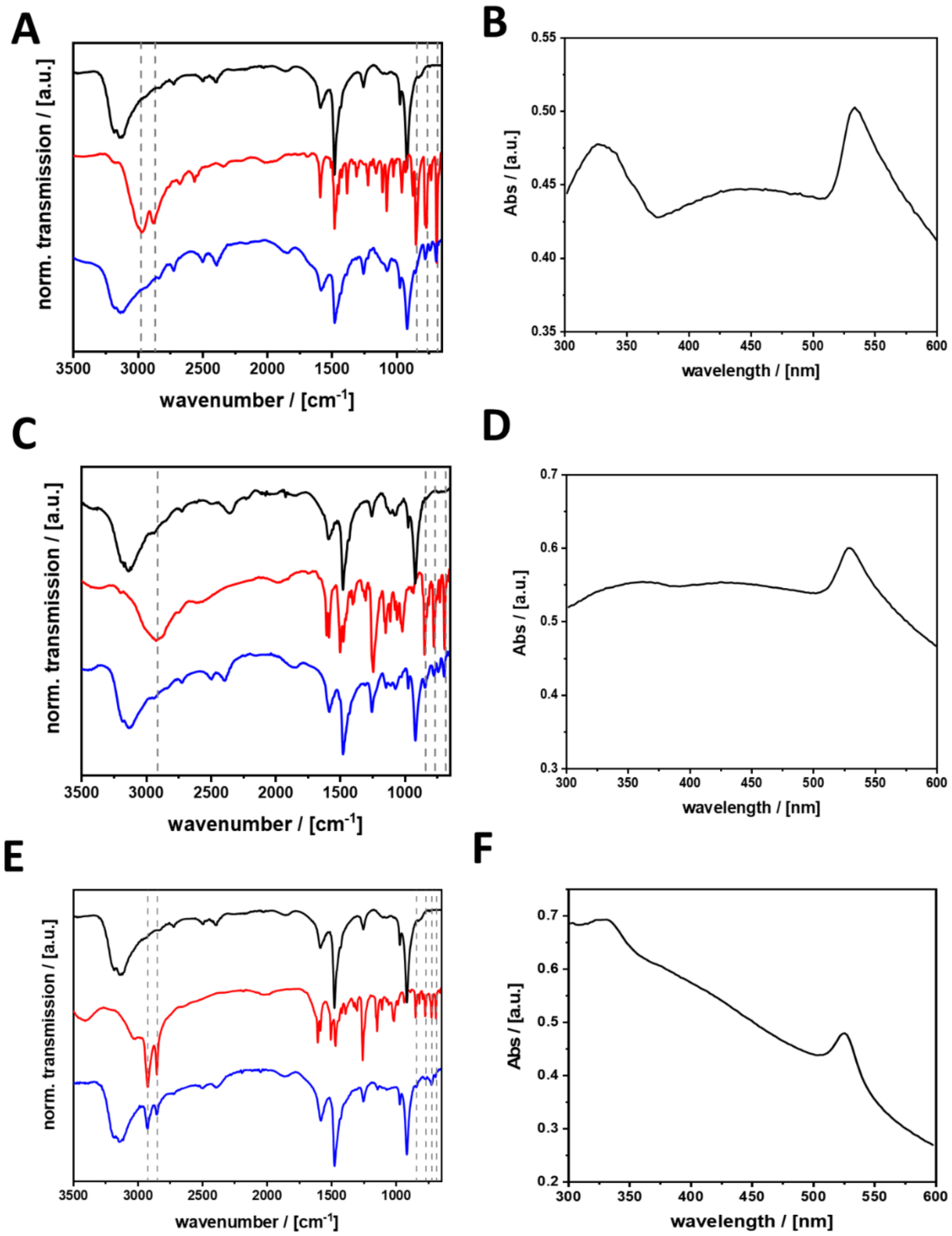

Figure S17: (A) IR spectrum of $AzoC_1$ (red), $CH_3NH_3PbBr_3$ (grey) and 3D-$AzoC_1$. (B) UV–vis absorption spectrum of 3D-$AzoC_1$ in toluene. (C) IR spectrum of $AzoOC_4$ (red), $CH_3NH_3PbBr_3$ (grey) and 3D-$AzoOC_4$. (D) UV–vis absorption spectrum of 3D-$AzoOC_4$ in toluene. (E) IR spectrum of $AzoOC_{12}$ (red), $CH_3NH_3PbBr_3$ (grey) and 3D-$AzoOC_{12}$. (F) UV–vis absorption spectrum of 3D-$AzoOC_{12}$ in toluene.

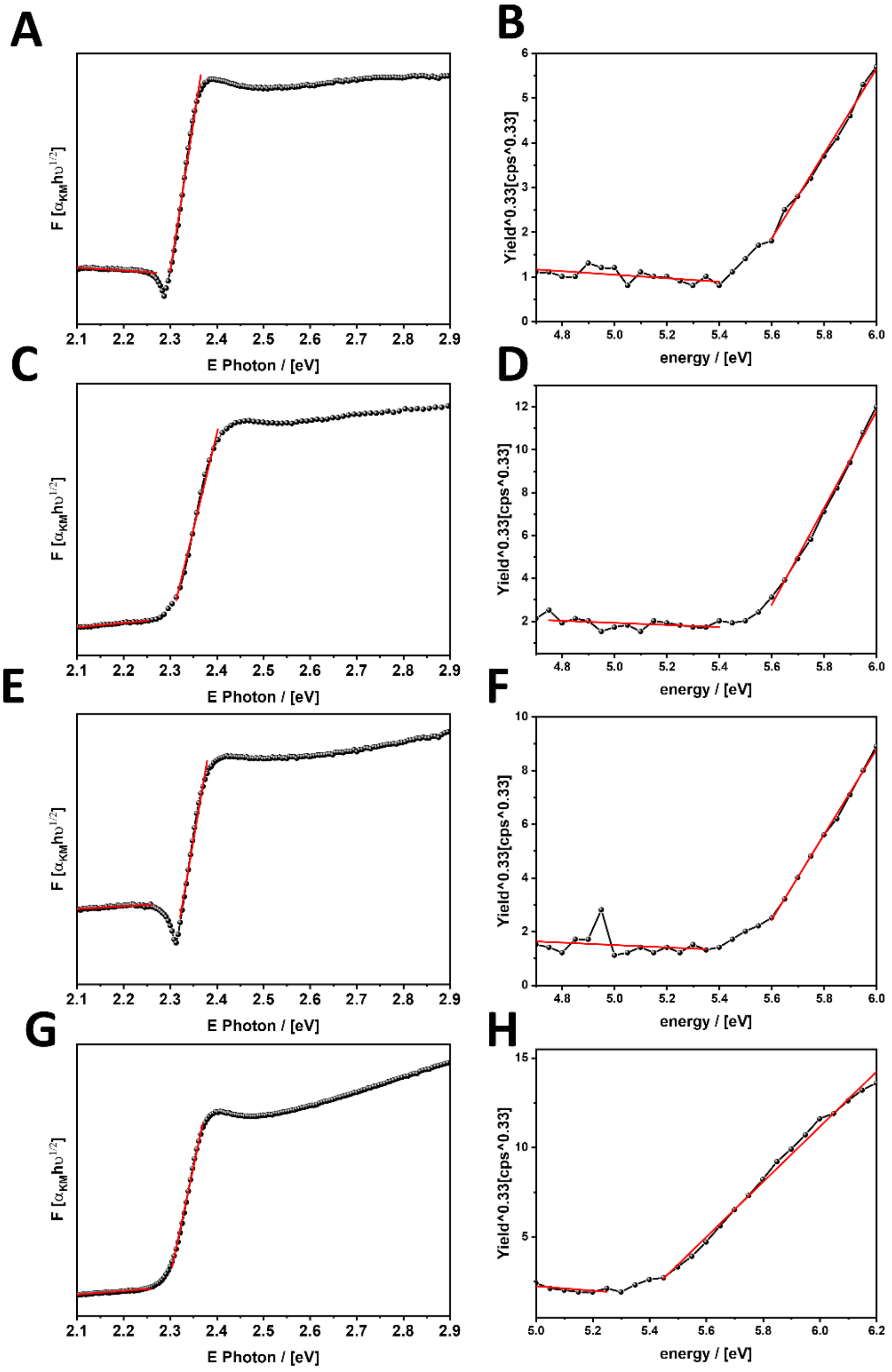

Figure S18: (A, B) UV–vis direct reflection and PESA measurement of 3D-AzoC$_1$. (C, D) UV–vis direct reflection and PESA measurement of 3D-AzoC$_2$. (E, F) UV–vis direct reflection and PESA measurement of 3D-AzoOC$_4$, (G, H) UV–vis direct reflection and PESA measurement of 3D-AzoOC$_{12}$. The intersection of baseline fit and slope fit gives the bandgap energy E_g or the relative energy E_{VB} of the valence band, respectively.

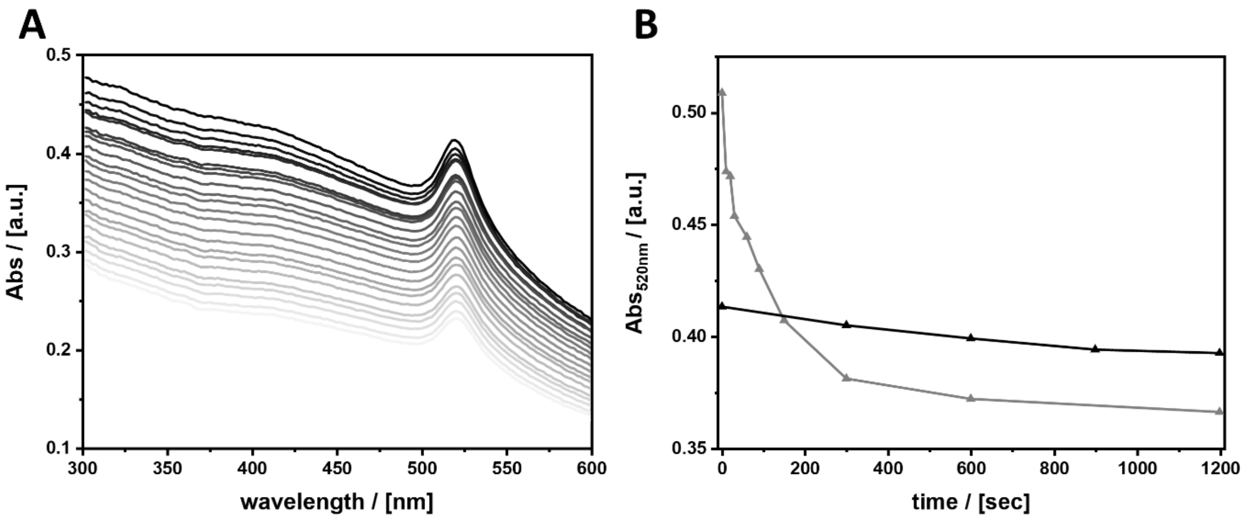

Figure S19: (A) UV–vis absorption spectra of a particle dispersion without irradiation recorded every 300 s. (B) Absorbance as a function of the time of dispersed particles without irradiation (black) and with irradiation (grey, spectrum see Figure 5 in the main manuscript). The dispersion is not stable over time, which is why we refer the azobenzene signal to the signal of the bandgap.

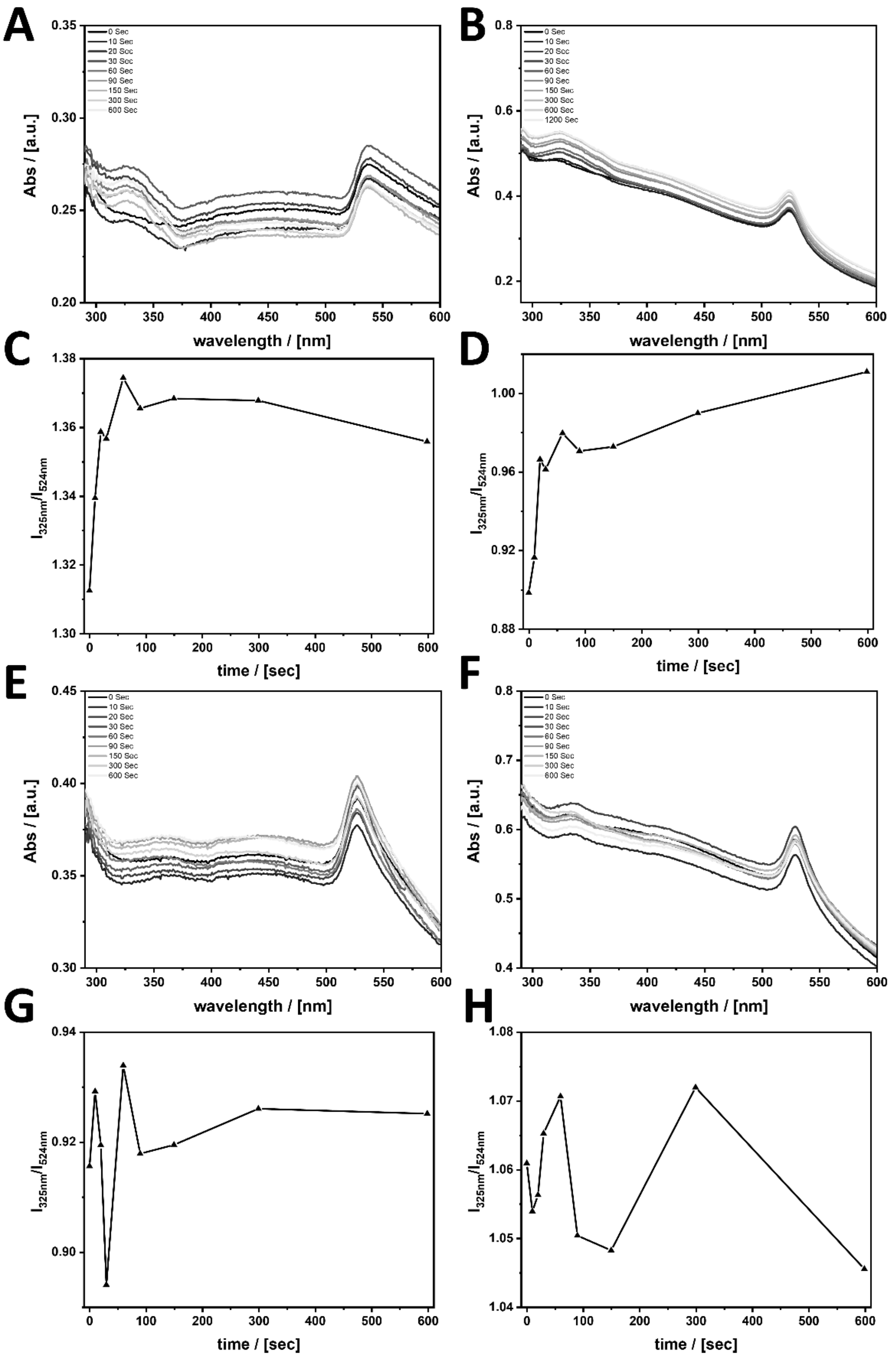

Figure S20: (A, B, E, F) UV–vis spectra of dispersions (irradiated for 5 min at 313 nm) of 3D-AzoC$_1$, 3D-AzoC$_2$, 3D-AzoOC$_4$ and 3D-AzoOC$_{12}$ in toluene while the particles are irradiated at 438 nm. (C, D, G, H) Intensity of absorbance at maximum of AzoC$_1$, AzoC$_2$, AzoOC$_4$ and AzoOC$_{12}$ divided by the intensity of the absorbance of the excitonic bandgap signal. As the dispersions were not stable they were referenced to the signal of the bandgap.

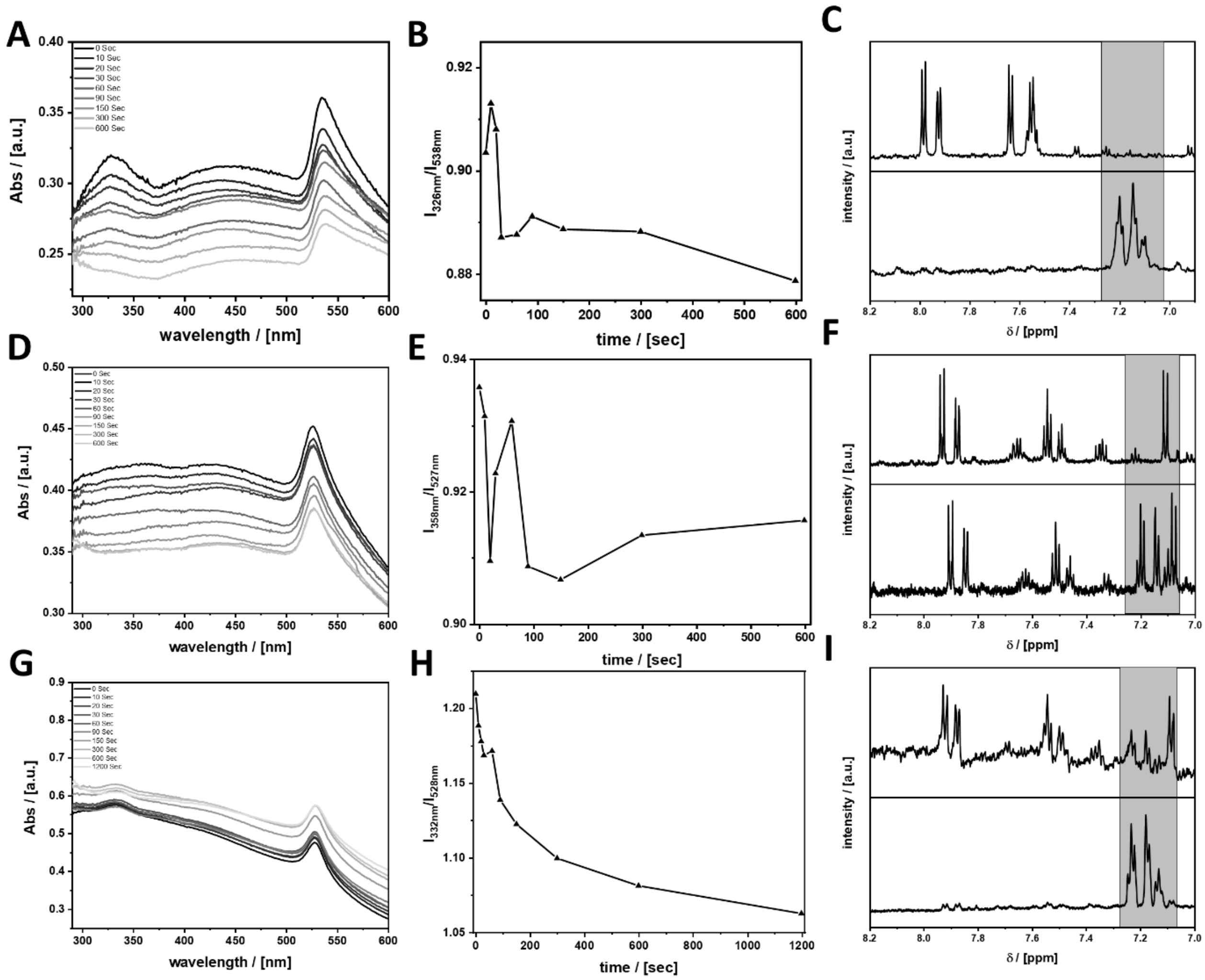

Figure S21: (A, B) UV–vis kinetic measurement of 3D-AzoC_1 in toluene over a period of time of 600 s. Because the dispersion is not stable, the absorbance of AzoC_1 at 326 nm is referenced to the absorbance of the excitonic bandgap at 538 nm and plotted as a function of the time. (C) ^{1}H NMR spectrum of dissolved 3D-AzoC_1 before (top) and after irradiation at 313 nm for 600 s (bottom). (D, E) UV–Vis kinetic measurement of 3D-AzoOC_4 in toluene over a period of time of 600 s. Because the dispersion is not stable, the absorbance of AzoOC_4 at 358 nm is referenced to the absorbance of the excitonic bandgap at 527 nm and plotted as a function of the time. (F) ^{1}H NMR spectrum of dissolved 3D-AzoOC_4 before (top) and after irradiation at 313 nm for 600 s (bottom). (G, H) UV–vis kinetic measurement of 3D-AzoOC_{12} in toluene over a period of time of 1200 s. Because the dispersion is not stable, the absorbance of AzoOC_{12} at 332 nm is referenced to the absorbance of the excitonic bandgap at 528 nm and plotted as a function of the time. (I) ^{1}H NMR spectrum of dissolved 3D-AzoOC_{12} before (top) and after irradiation at 313 nm for 1200 s (bottom).

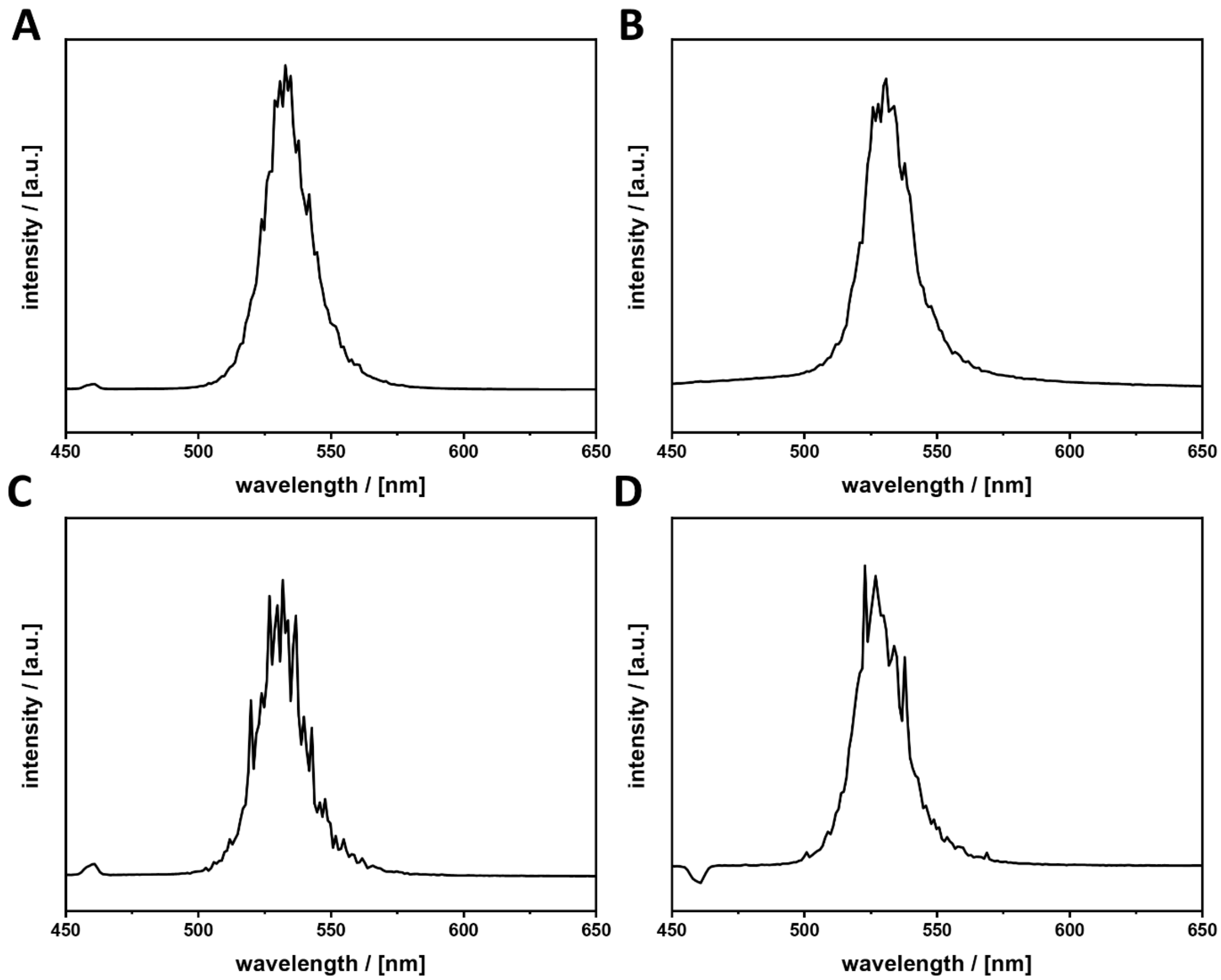

Figure S22: PL spectra of (A) 3D-AzoC1, (B) 3D-AzoC2, (C) AzoOC4 and (D) AzoOC12 dispersed in toluene, with λ_{exc} = 405 nm. To filter out the excitation wavelength a cut-off filter at 420 nm was used. Signals at 460 nm originate from toluene.

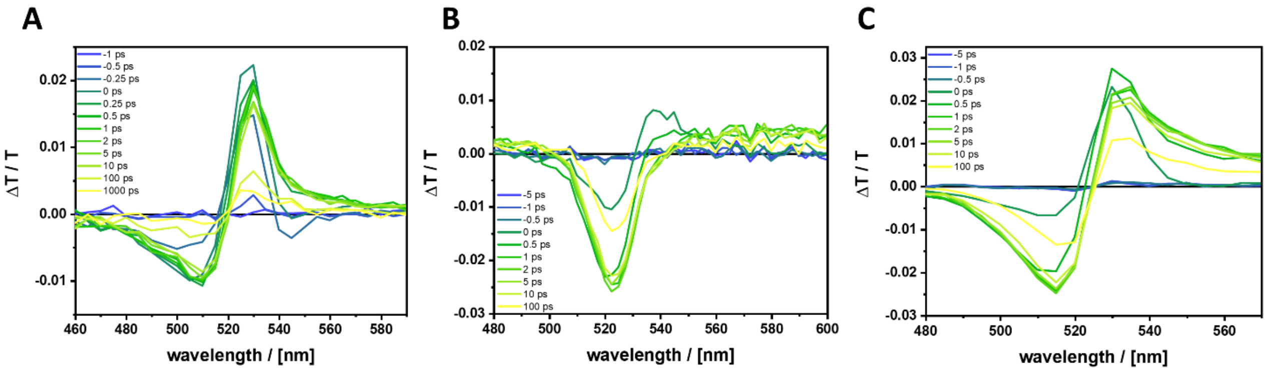

Figure S23: Transient absorption spectra of (A) 3D-AzoC$_1$, (B) 3D-AzoC$_2$ and (C) 3D-AzoOC$_{12}$. All samples were excited with a 398 nm pulse. Negative signals are obtained due to refractive index changes, which are caused by a change of the bandgap energy. Positive signals show a ground-state bleach, as the absorption at the bandgap decreases due to excited states.
